# Modeling Extreme Rapid Thermal Processing in Chemical Vapor Deposition Reactors for Catalytic Growth of Carbon Nanotubes

**DOI:** 10.1021/acs.iecr.5c04214

**Published:** 2025-12-15

**Authors:** Julia Aurenzi, Moataz Abdulhafez, Golnaz Najaf Tomaraei, Shervin Sammak, Mostafa Bedewy

**Affiliations:** 1 Department of Mechanical Engineering and Materials Science, 6614 University of Pittsburgh, 3700 O’Hara Street, Pittsburgh, Pennsylvania 15261, United States; 2 Department of Industrial Engineering, 6614 University of Pittsburgh, Pittsburgh, Pennsylvania 15261, United States; 3 Department of Chemical and Petroleum Engineering, 6614 University of Pittsburgh, Pittsburgh, Pennsylvania 15261, United States

## Abstract

Chemical vapor deposition (CVD) reactors with rapid thermal processing (RTP) capabilities provide unique advantages for controlling nanomaterial synthesis. Hence, understanding the dynamics and spatial distribution of the temperature inside them is key for process control as well as for engineering the resulting structure and properties. We use the finite element method (FEM) to model the spatiotemporal evolution of temperatures inside a custom-designed extreme multizone reactor for CVD of carbon nanotubes (CNTs) and graphene with RTP capabilities. Heat is primarily generated by 12 infrared (IR) lamps distributed both above and below a quartz tube in the reaction zones. Radiation is modeled by using the Monte Carlo radiation model. A catalyst-coated silicon chip is placed on a quartz support in the middle of the reactor. A thermocouple is modeled as a composite, where an area-weighted average of all the components was used to determine bulk properties. A mesh convergence study consisting of three refinements was carried out to ensure proper mesh size, leading to a model with 1.4 million elements. The model was then validated by comparing simulations to experimental data relating the infrared lamp power-to-temperature rise dynamics measured by the thermocouple. Results show that our model adequately captures the extreme temperature transience in the reactor and can hence be used to accurately explain the influence of boundary conditions on the spatial distribution of temperatures as well as the kinetics of heating of both the thermocouple and the catalyst-coated chip. Accordingly, the model is powerful for the design of new support wafer geometries and materials to precisely control the temperature distribution to achieve unprecedented spatial control in nanocarbon synthesis.

## Graphical abstract

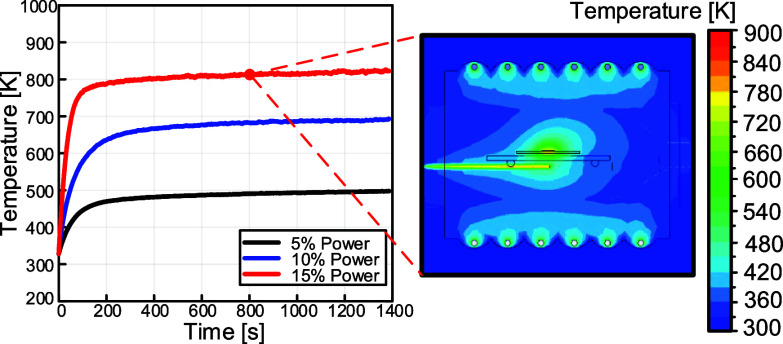

## 1 Introduction

Chemical vapor deposition (CVD) involves chemical reactions of gaseous precursors occurring both in the gas phase and on the substrate surface, leading to the formation of a thin solid film on the substrate. Catalytic CVD is a high-yield method widely used to produce carbon-based nanomaterials, such as carbon nanotubes (CNTs)^1–6^ and graphene^7^ from metal catalysts. Several factors must be considered when designing a CVD process and reactor, including process pressure and temperature, both in the gas-phase and at or near the deposition surface. Thermal energy is often used to activate reactions in CVD processes. Common heating methods include resistive heating, radio frequency (RF) induction, and infrared (IR) radiation.^8^ Thermal CVD reactors are classified as hot-wall and cold-wall reactors. In hot-wall reactors, the whole reactor volume can be considered a hot zone.^8^ The gas phase, reactor walls, and the substrate are all at the same temperature (or follow a determined gradient) in these reactors.^9^ For nanomaterial synthesis, these hot-walled reactors typically consist of a horizontal quartz tube chamber with the deposition zone inside a tube furnace.^8^

In cold-wall reactors, however, only the substrate is heated to a desired temperature, while the gas phase and reactor walls are maintained at ambient temperature or a temperature below the substrate.^9^ Rapid thermal processing (RTP) is a fast-heating technique that uses either transient lamp irradiation or rapid movement of the substrate in and out of the vicinity of a continuous heat source. The RTP technique has been combined with CVD to grow complex, multilayered structures by rapidly adjusting the temperature for each layer.^10^ Despite short processing times, RTP-based reactors face new challenges including substrate temperature measurement and control as well as temperature uniformity.^10,11^

Temperature measurement techniques in RTP-based reactors typically use thermocouples or pyrometers. Thermocouples are often embedded in a Si chip near the substrate,^10^ and due to their thermal mass may not capture the rapid nature of heating in RTP reactors. Another major issue is that the absorptivity of the Si chip changes over time due to material deposition. Thermocouples also have the issue of degradation with time due to intense thermal cycles. On the other hand, pyrometry is based on emitted radiation, which depends on both surface temperature and surface emissivity. The thickness of the deposited film as well as the back side roughness of the wafer affects the surface emissivity. Newer temperature measurement techniques are noncontact and are based on *in situ* monitoring of a wafer property that is influenced by temperature, such as in situ ellipsometry or laser interferometry for measuring optical properties.^10^ However, such techniques must have a fast response time to be suitable for rapid, dynamic processes.

Despite the availability of various reactor designs, most CVD reactors used for growing vertically aligned CNT (VACNT) forests feature a single reaction zone that is heated at slow rates (e.g., in a tube furnace). A key problem with such reactor designs is the thermal coupling of reaction conditions for the complex, molecular-scale physicochemical processes involved in forest growth. This coupling can lead to growth inconsistency and limited control over forest structure, density, and properties. One solution is to modify the reactor design to allow independent temperature control for the main processes: gaseous precursor decomposition, catalyst pretreatment, and CNT nucleation/growth. A summary of previous reactor design efforts to address the coupling problem is available in our earlier work.^12^ Briefly, several approaches have been developed to decouple the temperature of gaseous precursor reactions from that of catalyst pretreatment and CNT nucleation/growth: (1) adding a separate preheater for gas phase decomposition;^12–14^ (2) using a hot tungsten wire filament (>2000 °C) at a certain distance from the substrate to enhance gaseous precursor decomposition without increasing substrate temperature;^15–17^ (3) creating spatially controlled thermal zones for gaseous precursor reactions using independent heaters at different reactor locations;^18^ ^,^ ^19,20^ and (4) using a multizone furnace.^14^ In a smaller number of studies, decoupling the catalyst pretreatment temperature from the CNT nucleation/growth temperature has been achieved by using IR irradiation for rapid heating,^21,22^ or by rapidly moving the substrate in and out of the heated zone.^23^ In contrast, we use an extreme multizone RTP reactor with IR heating to achieve the complete decoupling of the temperatures of the three main processes to overcome challenges and limitations in CVD reactors.^11,12,24,25^

### 1.1 The Importance of Modeling for Custom-Designed Reactors

Since the early development of CVD processes and reactors, mathematical models have been used to relate the deposition performance to the reactor geometry and process conditions. Computational fluid dynamics (CFD) has been employed to reduce the time and cost of step-by-step development and iterative improvement of reactor prototypes.^26^ CVD is a highly complex process, making it difficult to perform holistic experimental studies. Modeling helps to identify the dominant processes that govern growth kinetics, material uniformity, and deposited film properties under varying reactor and substrate configurations. These models typically account for gas mixture flow, transport phenomena, chemical reaction pathways, reactor geometry, and the temperature of reactor walls and substrate.^26^

Modeling RTP-CVD reactors is particularly important due to challenges in experimental substrate temperature measurement, control, and uniformity.^10^ In our custom-designed reactor, the unique challenges are detailed here. First, the absorption, reflectivity, and emissivity of the thermocouple and the substrate are different, and the thermocouples are embedded in a quartz arm that is not in direct contact with the catalyst-coated silicon chip. As a result, closed-loop control signals used to regulate IR lamp power do not reflect the true substrate temperature. Additionally, transient temperature overshoot is commonly observed due to the high heating rates used in RTP. Heat conduction at high temperatures can help in faster relaxation of temperature to the steady state. However, the thermocouples may not reliably detect these rapid, nonuniform temperature transients due to their own thermal mass. Furthermore, while the design and properties of the chamber and heating system remain consistent, the design and optical properties of the support wafer may vary between experiments, leading to variations in radiative heat transfer, which affect the temperature uniformity across the catalyst chip and thus the uniformity of CNT forest properties. Therefore, modeling is critical to understanding the differences between the chip’s actual temperature profile and thermocouple readings. Importantly, spatial nonuniformities arising from geometric and dimensional inaccuracies or even density variations in as-grown macroscopic CNT structures, such as pillar,^27^ and millimeter-scale forests,^11^ are greatly sensitive to the actual spatial and temporal evolution of temperatures, which are hard to identify without modeling.

Thus, this work is unique and presents new methods for understanding the dynamics of surface reactions underlying the catalytic synthesis process in advanced CVD reactors. Here, the term advanced CVD reactors refers specifically to reactor architectures that depart from traditional single-zone, hot-wall tube furnaces by offering enhanced spatiotemporal control of temperature. These include systems that incorporate (i) rapid thermal processing via IR lamps, (ii) multizone top–bottom heating, (iii) independent temperature control for gas-phase precursor decomposition, catalyst pretreatment, and CNT nucleation/growth, and (iv) low-thermal-mass geometries that permit rapid ramps and fast thermal programming. Such reactor designs have emerged in recent years to overcome the coupling between gas-phase thermal chemistry and catalyst evolution that limits tunability in conventional CVD reactors.^11–15,21,22,24,25^ Hence, the present model, while tailored to our specific multizone RTP reactor, captures general thermal phenomena, including halogen lamp modeling, lamp/wafer coupling, thermocouple–wafer divergence, and spatial nonuniformity, which are also relevant to many CNT growth systems.

To strike a balance between the accuracy and practical implementation of a complete system model, we implement novel approaches that combine validated assumptions with multiphysics interactions. In particular, we model the IR lamps as hexagons instead of cylinders, as this proved to decrease the mesh size and computational run time without changing the temperature results. We also model the lamps as “voids”, meaning that the actual incandescent filaments are not present in the model. Instead, we make lamp-shaped holes in the fluid inside the IR chamber and apply our thermal boundary condition and radiative flux boundary condition to the outer fluid surface that would be in contact with the lamps if they were present. We use a validated relation between the lamp net radiative flux and lamp temperature to create accurate simulations for specific lamp powers, which has not been done previously.

Previous CNT CVD studies typically rely on a single embedded thermocouple or furnace set-point to represent the substrate temperature, without resolving the true temperature of the catalyst-coated chip or the spatial gradients across the wafer.^28–30^ In contrast, the present work explicitly distinguishes between the thermocouple temperature and the catalyst-chip temperature and directly models the thermal lag and spatial inhomogeneity between them. This distinction is critical in rapid thermal processing environments where transient and spatially nonuniform heating dominates the substrate behavior.

## 2 Methods

### 2.1 Description of the Extreme Custom-Designed RTP-CVD Reactor and Experiments

Figure 1 shows our custom-designed multizone RTP-CVD reactor. The resistive furnace preheats the process gases, which are supplied by a coiled injector. Mass flow controllers (MFCs) accurately control the transport rate of various gases. Adjacent to the preheater is the multizone infrared heater (highlighted in red), where catalyst pretreatment and CNT nucleation/growth occur. The reactor features three-zone control along the axis of the IR heater, with independent control over the top and bottom IR lamps in each zone. Three independent thermocouples are embedded in the quartz arm directly beneath the sample location. The signals from these thermocouples, fed into a digital temperature reader, are used in a closed-loop control system to regulate the lamp power in each zone. IR heating also illuminates the reaction chamber, allowing real-time monitoring and recording of CNT growth through the viewing port of the reactor by using a high-magnification CCD camera. Automated contrast adjustment is essential to prevent camera saturation during rapid temperature changes. Using image processing, catalytic lifetime and growth rates are quantitatively measured from the time evolution of forest height.^31^ A variety of automated dynamic recipes can be programmed, and their growth data are recorded for analytics.

**Figure 1:**
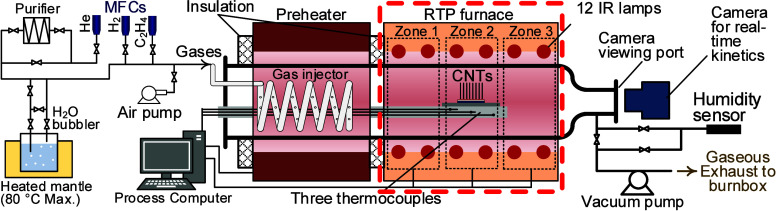
Schematic illustration of our custom-designed multizone RTP-CVD reactor. The RTP furnace, highlighted in red, is the main focus of our model.

#### Uniqueness of This Custom-Designed RTP-CVD Reactor

While previous attempts at customizing reactor design achieved partial temperature decoupling of the three main processes, complete decoupling of all three temperatures has only been achieved by our group using this custom-designed extreme multizone RTP-CVD reactor.^24^ In this unique design, the resistive preheater allows for the decoupling of the gaseous precursor decomposition. The multizone IR heater with independent control of the power to top and bottom IR lamps in each zone is capable of fast heating (>50 °C/s) and creating spatiotemporal temperature gradients. The low thermal mass of the quartz-based RTP system enables relatively high cooling rates, complementing the rapid heating capabilities. These unique features realize the decoupling of the temperatures of catalyst pretreatment and CNT nucleation/growth.

#### Description of Experiments

Recipes were designed to vary the IR lamp power in each zone and investigate its effect on the steady-state temperature of the preheater and each IR zone as well as the time it takes to reach the steady-state temperature. Before each experiment, the preheater and IR zones are at ambient temperature. The sample had a 1 nm iron catalyst film supported on a 10 nm alumina layer deposited on a Si chip with 300 nm thermal oxide. This sample is loaded in the recessed area of the substrate holder, which is positioned on the quartz arm. In each recipe, after the IR lamps are turned on and warmed up for a minute, the power to all three zones is manually set between 0 and 65%, with no closed-loop temperature control. The total flow rate was 1700 SCCM of He in all experiments. Once steady-state temperatures are reached in the preheater and all zones, the recipe is concluded and the reactor is cooled before starting the next experiment. Figure S1 in the Supporting Information shows the temperature profiles and steady-state temperatures of the center zone and preheater at various IR lamp power levels.

### 2.2 Modeling the Extreme RTP-CVD Reactor

Creating a computational fluid dynamics (CFD) model of an IR reactor presents several challenges. In general, determining a satisfactory time stepping scheme and mesh is always a factor in any CFD project, and these were especially important in our case. A model that includes radiative heat transfer adds much more complexity and thus many more factors to work out. Historically, the Monte Carlo radiation model has been most frequently used in simulations involving IR reactors, and this is the radiation method used here. Another necessary consideration in CFD modeling is how to model turbulence, which in our case involved researching and selecting the correct turbulence model in Ansys CFX. Because of the radiation-driven nature of the simulations, the choice of turbulence model was prioritized by simulation run time as opposed to turbulence resolution. As a result, the two-equation Shear Stress Transport (SST) turbulence model was chosen. Furthermore, convective heat flux was determined to be insignificant compared with the magnitude of radiative heat flux. This also justifies our decision to set our fluid domains to nonbuoyant. An additional set of challenges when creating a CFD model of a CVD reactor comes with small measurements of the geometry. For example, the catalyst-coated chip is very small and thus requires a highly refined and dense mesh with a large number of elements to capture the temperature distribution, and a larger number of elements in a CFD model greatly increases the computation time. Some parts of the model can be simplified to reduce the number of elements and computation time, such as omitting thermally irrelevant parts of the geometry, which will be discussed in detail in later sections.

In recent years, several studies have explored the modeling and simulation of IR reactors, and modeling the IR lamps seems to be one of the biggest challenges.^32–37^ Logerais et al.^32,33^ use the Monte Carlo method in a 3D CFD model of an IR reactor to calculate radiative heat transfer from the lamps, similar to our work. However, the geometry and thermal boundary conditions of the lamps differ from those of our model. Logerais et al.^32,33^ model the quartz bulb, tungsten filament, and nitrogen inside the lamp and apply temperatures specific to a certain lamp power percentage to the tungsten filament modeled as a cylinder in the center of the lamp. Jenkins et al.^34^ model only the filament of the lamps. Turner and Ash^35^ model both the filament and the quartz bulb, but the filament is treated as the primary source of heat generation. Jadachowski et al.^36^ present a 1D model where the IR lamps are infinitely long opaque cylinders and use the net radiation method to calculate the radiative heat flow in the IR zone. Yu et al.^37^ show that their quartz lamps could be modeled as a slat with the same temperature radiation source.

Modeling radiative heat transfer has also been a heavily researched topic in recent years. In modeling radiative heat transfer in IR chambers, use of the Monte Carlo method is the most common.^32,33,35^ However, some others use different methods. For example, Abigail Wacher^38^ uses shape factor theory to calculate the radiative heat transfer in a rapid thermal processing system. Jenkins et al.^34^ use the Discrete Ordinates (DO) model in Ansys Fluent, and Yu et al.^37^ use a finite volume method algorithm to capture the radiative heat transfer from quartz lamps in their system. Chao et al.^39^ study the effect of lamp radius on thermal stresses that contribute to wafer temperature nonuniformity focusing on edge effects. They treat the problem as a 1D plane stress model using a fully implicit finite difference method. They quantify the effects of radiative heat transfer using the law of radiative heat transfer to obtain view factors between the lamps and the wafer, where the tungsten-halogen lamps are modeled as flat blackbody radiation sources.

#### Material Properties in Our Computational Modeling

One of the first steps in setting up a simulation in Ansys CFX, Release 2021 R2, is to define the material properties used in each domain. Several of these materials were already present in the Ansys material library, such as Air at STP and He at STP; however, the rest of the materials used in the model needed to be user-defined. The material properties for gold, silicon, and fused silica (quartz) are well documented and easily obtained. For example, density, specific heat, and thermal conductivity values at 300 K were obtained from Fundamentals of Heat and Mass Transfer.^40^ However, defining the material properties for the thermocouple (TC) was more complex and will be discussed in detail in later sections. Table S1 in Supporting Information Section S2 provides a detailed description of all the material properties required to define each material in Ansys CFX, Release 2021 R2.

#### Governing Equations

Ansys CFX solves the unsteady Navier–Stokes equations in their conservation form, according to the Ansys CFX Solver Theory Guide.^41^ These equations include the transport equations: continuity (eq 2.1), momentum (eq 2.2), the stress tensor τ and its relation to strain rate (eq 2.3), total energy (eq 2.4), and the total enthalpy *h*_tot_ and its relation to static enthalpy *h*(*T*, *p*) (eq 2.5).

$$\frac{\partial \rho }{\partial t}+\nabla \times (\rho U)=0$$ 2.1

where ρ is density and *U* is the vector of velocity (*U*_*x*,*y*,*z*_).

$$\frac{\partial (\rho U)}{\partial t}+\nabla \times (\rho U⊗U)=-\nabla p+\nabla \times \tau +S_{M}$$ 2.2

where *p* is static (thermodynamic) pressure, τ is shear stress (or subgrid scale stress) molecular stress tensor, and *S*_M_ is the momentum source term.

$$\tau =\mu \left(\nabla U+(\nabla U{)}^{T}-\frac{2}{3}\delta \nabla \times U\right)$$ 2.3

where μ is molecular (dynamic) viscosity, *T* is static (thermodynamic) temperature, and δ is the identity matrix or Kronecker Delta function.

$$\frac{\partial (\rho h_{\mathrm{tot}})}{\partial t}-\frac{\partial p}{\partial t}+\nabla \times (\rho Uh_{\mathrm{tot}})=\nabla \times (\lambda \nabla T)+\nabla \times (U\times \tau )+U\times S_{M}+S_{E}$$ 2.4

where *h*_tot_ is specific total enthalpy, λ is thermal conductivity, and *S*_E_ is the energy source term.

$$h_{\mathrm{tot}}=h+\frac{1}{2}U^{2}$$ 2.5

where *h* is specific static (thermodynamic) enthalpy.

#### Selecting a Radiation Model

Ansys CFX offers several radiation models: Monte Carlo, P1, Rosseland, and Discrete Transfer. Detailed descriptions of each model are provided in Supporting Information, Section S3. In the early stages of the project, the P1 radiation model was used, but it was decided that Monte Carlo would better suit our needs. This decision was based on several factors. First, Monte Carlo can be used in both solid and fluid domains, and it was determined that we would need to use radiation in all domains; therefore, this was the best option. Monte Carlo can also be used if the medium is optically thick or thin, while the P1 model should only be used if the medium is optically thick. Optically thick implies that the medium is opaque, meaning that the average photon cannot pass through the medium without absorption, and a fluid will absorb and then re-emit radiation that passes through it. Optically thin implies that the medium is transparent, and radiation only interacts with boundaries of the domain. The Monte Carlo model also includes both surface-to-surface and participating media transfer modes for all domains. As described in the Ansys CFX Solver Modeling Guide,^42^ the surface-to-surface mode ignores volumetric emission, absorption, and scattering, even if these values are specified in the properties of the material assigned to the respective domain. The participating media transfer mode does the opposite, meaning that the domain material will emit, absorb, or scatter radiation. The surface-to-surface model is used in all fluid domains, the paddle, and the tube. This is because these are not the main subject of the simulations; therefore, radiation should just pass through these domains. The participating media model is used in the catalyst chip, IR chamber walls, and thermocouple domains, as these are the main focus of the simulation, and we need to see the effects of radiative heat transfer on these parts. When the Monte Carlo model is used, one must also select an appropriate number of histories. After several simulations where various numbers of histories were used, it was decided that the best number of histories for each domain was 1,000,000. Though this number is large and it is known that a higher number of histories leads to a longer run time, this number allowed us to capture the most accurate simulation results and was therefore necessary.

#### Model Simplifications and Boundary Conditions

A schematic of the IR chamber as modeled in Ansys CFX is shown in Figure 2, with each part of the geometry labeled. A detailed model of the tray configuration is provided in Supporting Information Section S4, Figure S2. The rest of this section describes how each part was modeled and what boundary conditions were applied. Detailed outlines of the domain and interface settings are provided in the Supporting Information Section S5, in Tables S2–S4 with letter labels corresponding to those in Figure 2.

**Figure 2:**
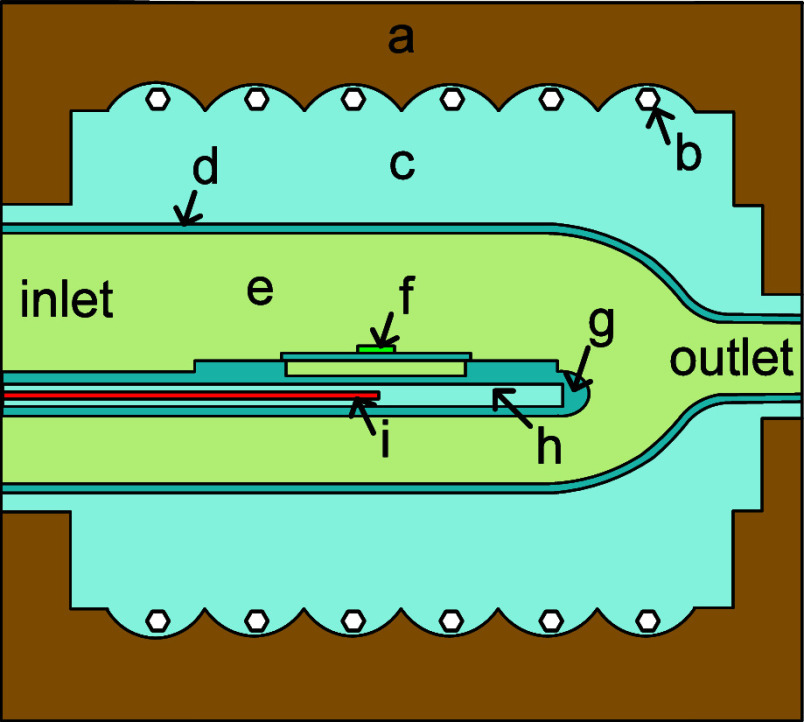
(a) IR chamber walls, (b) IR lamps, (c) IR chamber fluid, (d) reactor process tube, (e) reactor process tube fluid, (f) catalyst-coated Si chip (g) paddle, (h) paddle inner fluid, and (i) thermocouple.

#### Key Simplifications

When creating a CFD model, making simplifications that cut down on the number of elements and thus computational time is a crucial step. It was decided to exclude the fans, cooling lines, and preheating devices from the CFD model of the reactor. Instead, the preheater was accounted for using a boundary condition of 328 K as an initial temperature for all domains instead of starting at room temperature. Neglecting the cooling lines and fans potentially has an effect on the heating rates and steady-state temperatures of both the catalyst chip and the thermocouple, which is discussed in more detail in the heat loss investigation section. Another simplification was neglecting the detail above the arches in the IR chamber of the reactor, where the lamps are located, along with changing the lamps from cylindrical to hexagonal, which will be discussed in detail in later sections.

#### IR Chamber Walls

The IR chamber walls in the CVD reactor are gold coated. In the model, gold is used as the bulk material for this geometry. The outer surfaces of the chamber walls are modeled as fixed-temperature surfaces (328 K) that are opaque, with an emissivity of 0.02 and a diffuse fraction of 0. These emissivity and diffuse fraction values are chosen to represent mirror-like behavior, consistent with the gold coating of the actual reactor walls, which reflects radiative heat from the IR lamps. The inner surfaces of the IR chamber walls that are in contact with the fluid inside the chamber are modeled by using a fluid–solid interface in Ansys CFX. In this type of interface, there are settings for mass and momentum, wall roughness, and heat transfer. In our case, mass and momentum were set to no slip wall, thus using the no slip boundary condition. The wall roughness was set to a smooth wall, as the inner chamber walls are smooth in reality. Lastly, the heat transfer was set to a conservative interface flux, which is the only available option for this type of interface.

#### IR Chamber Fluid

In the physical CVD reactor, the fluid inside the IR chamber surrounding the lamps and process tube is air. Therefore, in the model, this domain’s material is set to air at STP. Since this is a fluid domain, a turbulence model must be selected. In our case, the Shear Stress Transport (SST) model was determined to be the most suitable. For the purpose of our simulations, the inlet and outlet of this domain are set to walls with the same settings as the IR chamber walls. The IR lamp boundary conditions are also set in this domain, since the lamps are modeled as voids and are not actually present in the geometry, which will be discussed in more detail in the following section.

#### IR Lamps

Several lamp geometries were created and tested, but the most studied geometries were cylindrical and hexagonal shaped lamps. The shape of the lamps was changed from cylindrical to hexagonal to reduce the mesh around the lamps. It was noticed that the cylindrical shape caused a much finer mesh than necessary due to capturing the curves of the cylindrical shape; therefore, hexagonal lamps were created. When creating the hexagonal lamps from the cylindrical lamps, the surface area must be the same, so that the results from both the cylindrical and hexagonal lamps could be compared to make sure that the results were the same. It was known that the 5 mm-diameter cylindrical lamps, which the hexagonal lamps were based off of, had a surface area of 2960.95 mm^2^, so the dimensions of the hexagonal lamps were determined based off of this. The hexagonal lamps have a side length of 2.6212 mm, which yields a 2960.9263 mm^2^ surface area, which is very close to the cylindrical lamp surface area.

The hexagonal lamps eliminated the need for the curves to be captured and allowed the mesh to be refined by using face meshing on each of the six sides of the hexagon. A mesh convergence study was performed to determine the best face meshing element size. The starting point had an element size of 2.6212 mm, which is the length of one of the six sides of the hexagon. The first refinement divided this face meshing by 1.5, giving an elemental size of 1.7474 mm. The second refinement divided the face meshing by two with an element size of 1.3106 mm. The third refinement divided the face meshing by 1.4 creating an element size of 1.8723, and last the fourth refinement divided the face meshing by 2.25 creating an element size of 1.165 mm. It was determined that the third refinement with an element size of 1.8723 mm on each face of the hexagonal lamps produced the best results, while still significantly lowering the number of elements from the cylindrical lamp model. This lamp meshing was then adopted for all of the following simulations where the hexagonal lamps were used. Figure 3 shows a comparison of the mesh around the original cylindrical lamps and the hexagonal lamp mesh refinements. These meshes and all others mentioned in this work were generated using the Fluid Flow (CFX) Analysis System in Ansys Workbench, Version 2021 R2.

**Figure 3:**
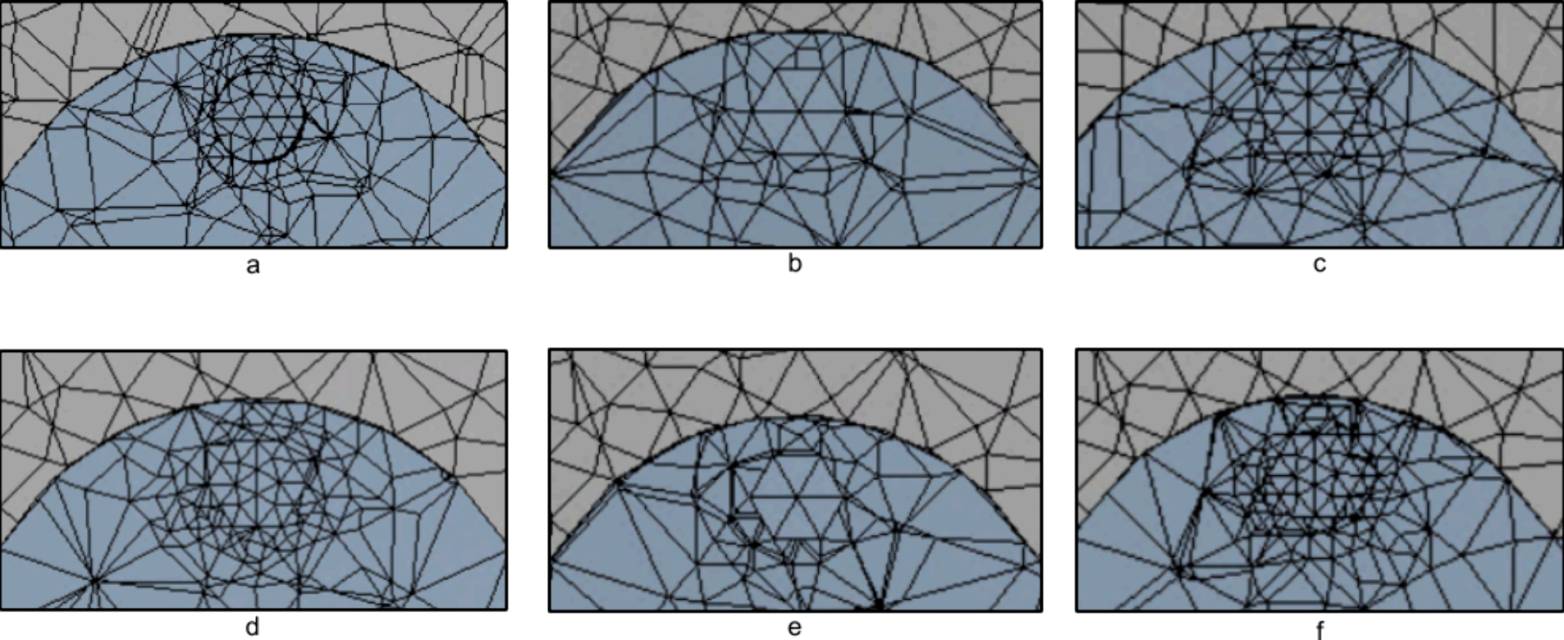
(a) Original cylindrical lamp mesh, 574,978 elements. (b) Original hexagonal lamp mesh, face meshing element size 2.6212 mm, 275,719 elements. (c) Hexagonal lamp mesh refinement one, face meshing element size 1.7474 mm, 400,441 elements. (d) Hexagonal lamp mesh refinement two, face meshing element size 1.3106 mm, 497,519 elements. (e) Hexagonal lamp mesh refinement three, face meshing element size 1.8723 mm, 327,827 elements. (f) Hexagonal lamp mesh refinement four, face meshing element size 1.165 mm, 555,186 elements. Images used are courtesy of ANSYS, Inc.

It should be noted that all lamps are modeled as “voids” to reduce the total element count in the model. The lamps are physically present in the original geometry and are then removed before the model is imported into CFX. This creates lamp-shaped holes in the fluid area inside the IR chamber. The lamp boundary conditions are then applied to the faces of the fluid that would be touching the lamps if they were present. A fixed temperature and radiation source of isotropic radiation flux are applied to these faces to simulate the specific lamp power. The values for lamp temperature and flux are described in detail in Section 3.

#### Reactor Process Tube

In the reactor, the process tube is made of fused silica; thus, this is the material that this domain is set to in the simulations. Determining the absorption coefficient of this material was the main challenge; many values were tested, and it was decided that a value of 0.1 1/m yielded results that most closely matched experimental data. The ends of the process tube are set to the same boundary conditions as the IR chamber walls since they are in contact with these walls. A fluid–solid interface is defined at the outer wall of the process tube, which is in contact with the IR chamber fluid. Here, both the heat transfer and thermal radiation options are set to a conservative interface flux. While there are other options available for this interface, conservative interface flux was determined to be the best option, as it implies that the heat transfer or thermal radiation will flow between that boundary and the boundary on the other side of the surface.^42^ Therefore, this allows heat and radiation to flow from the fluid surrounding the tube to the process tube.

#### Reactor Process Tube Fluid

The fluid inside the process tube is set to helium at the STP, matching the helium that is injected into the process tube in the real CVD reactor. Again, the SST is used as the turbulence model. Here, there are an inlet and an outlet. The inlet is set to a normal speed of 0.0062 m/s, calculated by dividing the 1700 SCCM He flow rate by the inlet diameter with a static temperature of 328 K. The outlet has a static pressure of 1 atm. The fluid–solid interface between the process tube and the process tube fluid is again set to a conservative interface flux.

#### Catalyst-Coated Silicon Chip

In reality, the chip is located on top of the paddle and is made of silicon; therefore, the material for this domain is set to that. The mesh of the chip is very fine, since we want to capture the temperature distribution on the chip as accurately as possible. There is a fluid–solid interface present between the chip and the fluid inside the process tube, where the heat transfer is set to a conservative interface flux. The thermal radiation, however, is set to opaque with an emissivity of 0.9 and a diffuse fraction of 1. These values were determined through several simulations. This does not quite represent a blackbody, which would have an emissivity of 1, but is very close and accurately represents the silicon wafer.

#### Paddle

The paddle in the reactor is made of fused silica and, therefore, is modeled as such. As with other solids, the end of the paddle that is not inside the reactor and is in contact with the IR chamber walls has the same settings as those of those walls. The catalyst-coated chip rests on the paddle, so there must be a solid–solid interface defined there, and a conservative interface flux is used for both heat transfer and thermal radiation.

#### Paddle Inner Fluid

The fluid inside the paddle is set to air at the STP, as the inside paddle fluid in the reactor is also air. This fluid does not have an inlet, and the end of the fluid that is not inside the reactor is again set the same way as the IR chamber walls. Since this is a fluid domain, the turbulence model is again set to SST. The interface between the solid paddle and fluid inside the paddle is again set to conservative interface flux for both the heat transfer and thermal radiation options.

#### Thermocouple

The thermocouple arrangement inside the reactor consists of three K-type thermocouples that start at the entrance of the process tube. The ends of these thermocouples are staggered by approximately 1 in., where the middle length thermocouple ends near the middle of the silicon chip. Each thermocouple consists of a Pyrosil D sheath, MgO insulation, one thermoelement made of alumel, and one thermoelement made of chromel. Since the model was already very complicated before the thermocouple was introduced, it was decided to model the thermocouple as one bulk material instead of modeling each of the elements. It was also decided to model just one thermocouple instead of all three. The thermocouple is surrounded by air space inside the paddle, and the end of the thermocouple is just beneath the center of the silicon chip. A schematic of this is shown in Figure 4.

**Figure 4:**
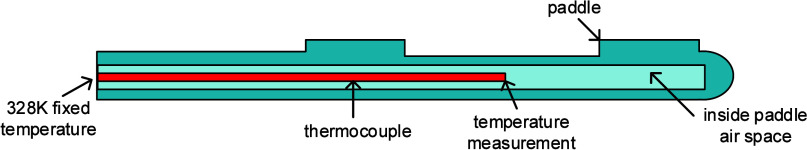
Thermocouple schematic.

## 3 Results and Discussion

### 3.1 Thermocouple Modeling Results

The most challenging aspect of modeling a thermocouple was determining its material properties. It was known that the thermocouple was type K, made up of a Pyrosil D sheath, MgO insulation, one wire made of alumel, and one wire made of chromel. The material properties for all components except the Pyrosil D sheath were well documented. As stated previously, it was decided to model the thermocouple as one solid material instead of modeling each individual component as that would add too much complication to an already detailed model. To do this, an area-weighted average of the material properties was calculated by using a diagram of a type K thermocouple. The percentage of the total thermocouple area for each component was calculated, and for each material property (density, specific heat, thermal conductivity, and molar mass), the component value was multiplied by its percentage and then added together to compute the effective material property. This approach was also used by Nakos^43^ for determining thermocouple material properties in the same way.

Once the initial set of thermocouple material properties were obtained, several simulations were completed at the same lamp power to test each material property. One property was changed at a time, and simulation results were compared to experimental data to determine which value was the most accurate. Emissivity values of 0.46, 0.66, and 0.86 were tested, based on values reported by Nakos^43^ and Brundage et al.^44^ Temperature vs time and temperature vs length plots were evaluated, and it was determined that 0.46 was the best emissivity value, as shown in Figure 5.

**Figure 5:**
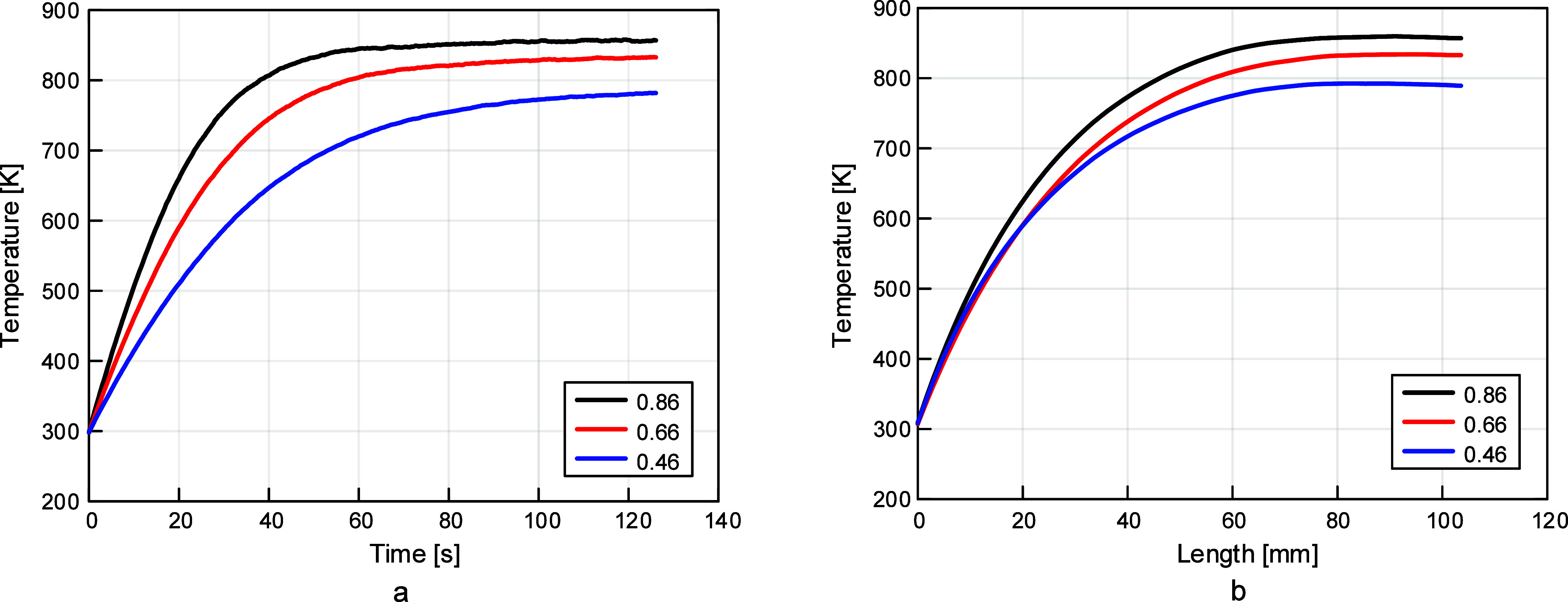
Thermocouple emissivity investigation results: (a) temperature vs time and (b) temperature vs length. Temperature was shown to increase with emissivity and an emissivity value of 0.46 produced results closest to experimental data.

The next material property investigated was the density. Values of 5880 and 6576 kg/m^3^ were tested, and it was determined that 6576 kg/m^3^ was the most accurate value, as shown in Figure S3 in the Supporting Information. The value of 5880 kg/m^3^ was obtained from Brundage et al.,^44^ and 6576 kg/m^3^ was calculated using the area-weighted average method mentioned above.

The specific heat of the thermocouple was also investigated. The calculated value was 618 J/kgK, and 696 J/kgK from Brundage et al.^44^ was also tested. As shown in Figure S4 in the Supporting Information, it was determined that the 618 J/kgK value was the most accurate.

The thermocouple material property that was investigated most extensively was the thermal conductivity. The originally calculated value was 67 W/mK, and values of 1.7 and 30 W/mK were also tested. The thermal conductivity of 1.7 W/mK came from Brundage et al.,^44^ and 30 W/mK was a chosen value between 1.7 and 67 for the purpose of testing. Neither of these values was satisfactory, so thermal conductivity was recalculated using new values but the same area-weighted average method. The new values for each component were obtained from Yilmaz.^45^ It was decided that these values closely represented our thermocouple because the sheath material in Yilmaz’s work is Inconel 600, and ours is Pyrosil D, and they both consist of about 70% nickel and 20% chromium. The insulation material used in Yilmaz^45^ is MgO as well as ours, and their thermoelements are alumel, while ours are alumel and chromel. Using thermal conductivity values for each element from Yilmaz,^45^ a new area-weighted average was created, yielding a new thermal conductivity of 8.67 W/mK. From Figure 6, it is evident that this thermal conductivity value produces the best results representing reality. While the temperature vs time plots would indicate that each of these tested values are relatively the same, plotting temperature vs length for a given point in time shows that 8.67 W/mK yields the most uniform temperature results. The importance of this spatial uniformity in thermocouple temperature measurement is explored further in Section 3.4 below.

**Figure 6:**
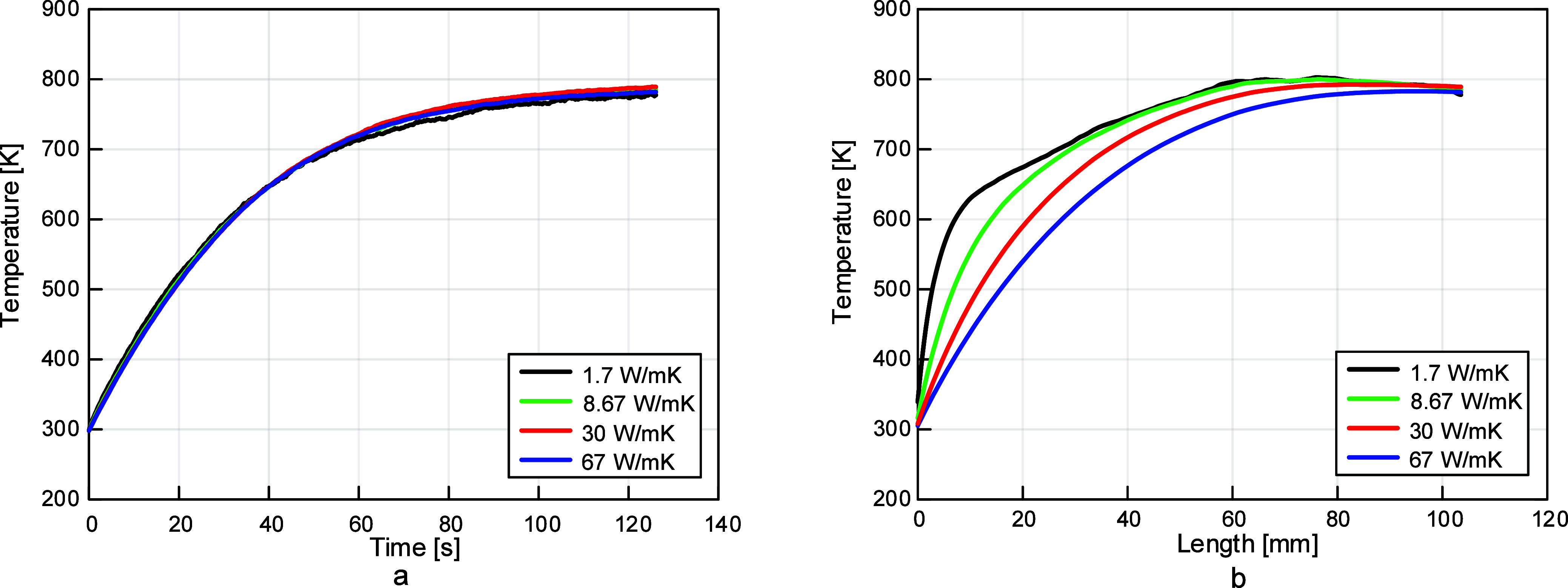
Results of the thermocouple thermal conductivity investigation, (a) temperature vs time and (b) temperature vs length. The value of 8.67 W/mK was determined to be the best value as this yields the most uniform temperature results along the length of the thermocouple.

It is important to note that the thermocouple assembly in our reactor is not a single homogeneous material but a composite structure consisting of a Pyrosil D sheath, MgO insulation, and chromel–alumel conductors. For this reason, the appropriate thermal conductivity is a cross-sectional area-weighted composite value rather than the conductivity of any individual component. We demonstrate that the value of 8.67 W/m·K, obtained by applying this composite approach using material data reported by Yilmaz,^45^ provides the most accurate thermophysical constants for Inconel-based sheathed thermocouples. Lower values such as 1.7 W/m·K, although reported in Brundage et al.,^44^ correspond to fire-exposed, large-diameter thermocouples and do not reflect the physical reality of our geometry. This explains why simulations using 1.7 W/mK produce unrealistic temperature gradients along the thermocouple length, whereas the composite value of 8.67 W/mK yields a physically consistent, smoothly equilibrated temperature profile.

### 3.2 Mesh Convergence

To ensure that the catalyst chip temperature results were independent of the mesh size, a mesh convergence study was conducted. The main focus was the area of the fluid inside the process tube that came into contact with the chip. Face meshing was performed on the six faces of the areas that came into direct contact with the six faces of the chip. The first refinement changed the face meshing element size from 0.5 to 0.3 mm, the second changed the size to 0.175 mm, and the third and final refinement changed the face meshing element size to 0.1 mm. Each refinement more than doubled the number of elements in the fluid inside the process tube. Nothing else was changed in the simulations to ensure that the differences in chip temperature were solely due to these refinements, and all simulations were conducted at the same lamp power. It was determined that the second refinement was sufficient since there was almost no change in chip temperature from refinement two to refinement three, as shown in Figures 7 and 8. A mesh convergence study was not necessary for the thermocouple, as the temperature values were independent of mesh size and time stepping and the values did not change if the time step or mesh size was changed.

**Figure 7:**
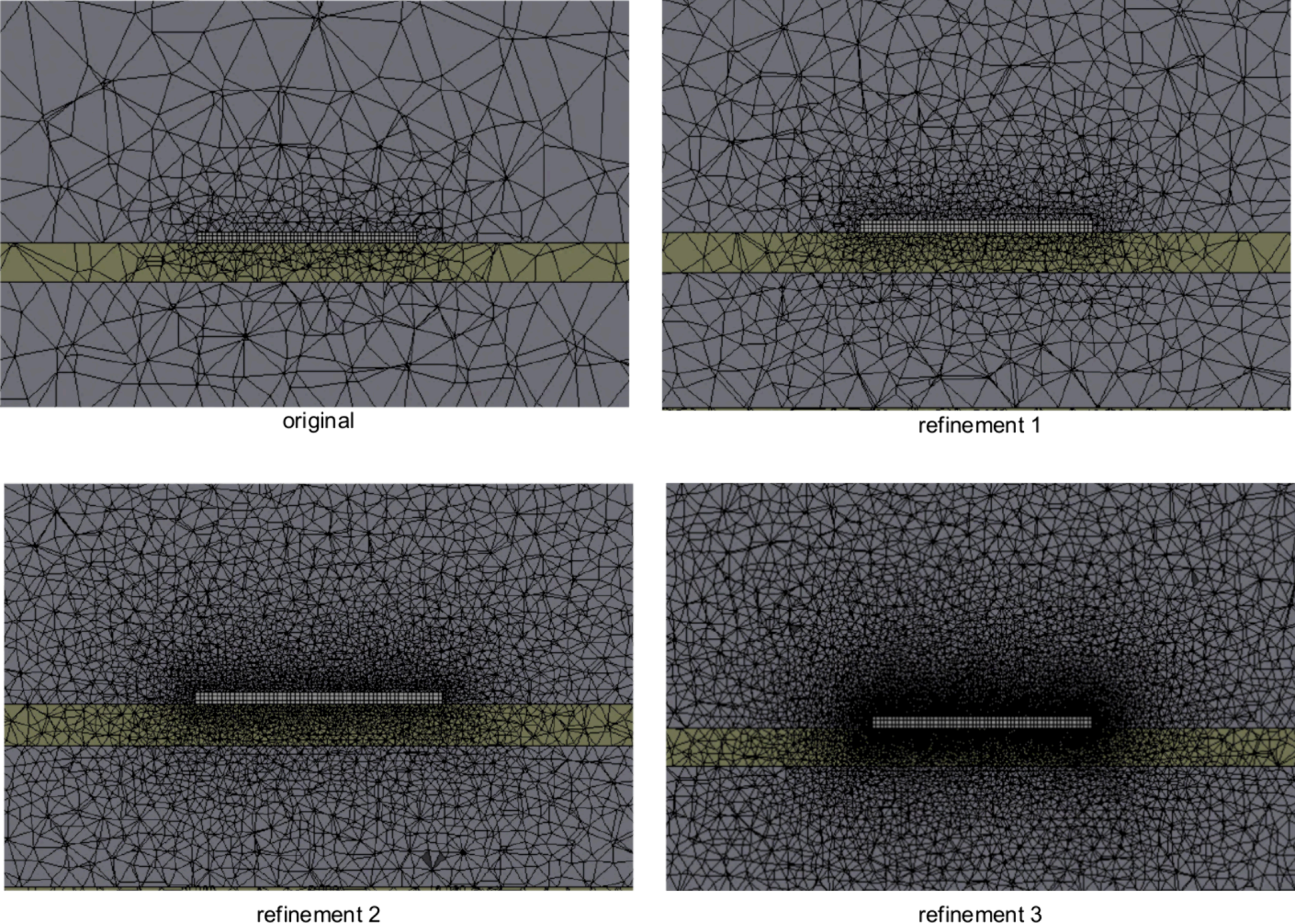
Mesh refinements, images used courtesy of ANSYS, Inc.

**Figure 8:**
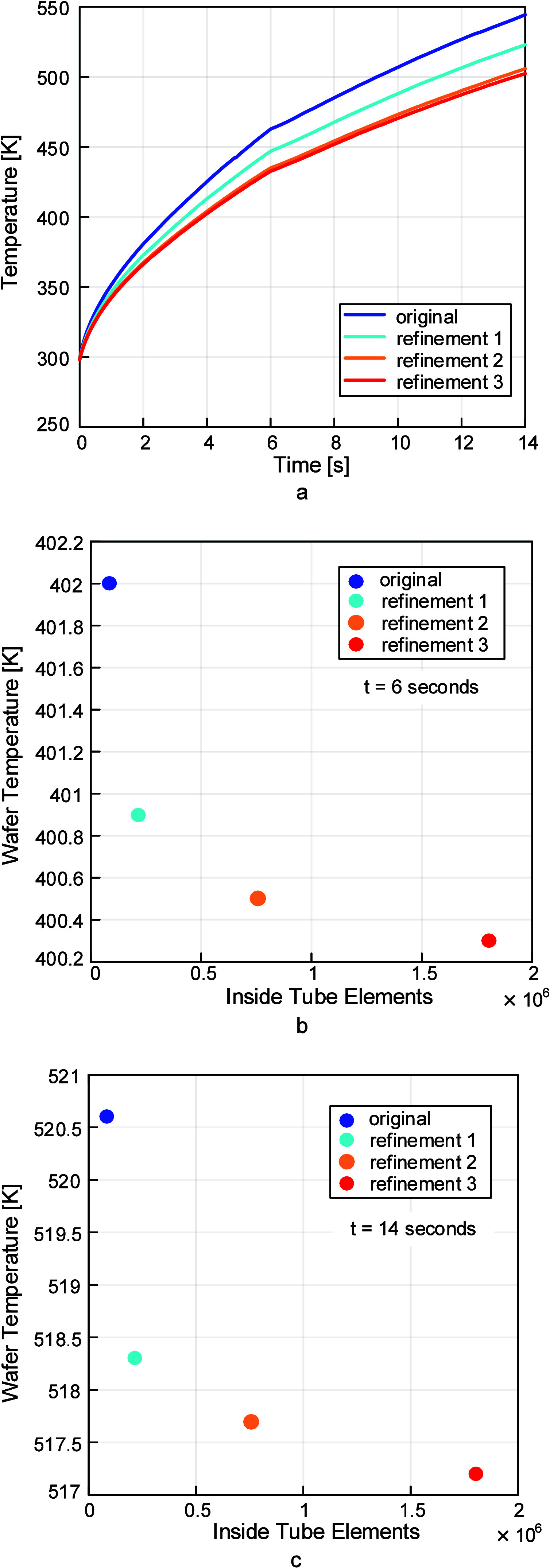
(a) Mesh refinements are shown as temperature vs time, demonstrating that the temperature change from refinement 2 to refinement 3 was small. The chart in (b) shows the chip temperature at 6 s for each of these refinements, and that in (c) shows the same for 14 s. From (b) and (c), it is evident that there is a large increase in elements in the inside tube domain and only a small difference in temperature, meaning that refinement 2 is sufficient and will have a significantly lower computation time than refinement 3.

### 3.3 IR Lamp Modeling and Experimental Validation of Model

Determining accurate thermal boundary conditions for the IR lamps was a central challenge in this work. To define the lamp surface temperature and the corresponding radiative heat flux applied to each lamp boundary in the CFD model, we used the experimentally measured envelope temperature data reported by Coaton for tungsten–halogen lamps with fused-quartz envelopes.^46^ In Coaton’s terminology, the envelope temperature refers to the temperature of the outer quartz wall of the lamp, measured directly using thermocouples or infrared pyrometry. It is not the filament temperature. This distinction is important because it is the envelope surface and not the tungsten filament that governs the radiative boundary condition in our model of the reactor.

Coaton provides envelope temperature measurements at four discrete electrical power inputs (389, 715, 1124, and 1412 W). Within this operating range, the envelope temperatures are nearly collinear when plotted against the input power. For this reason, and strictly as a practical interpolation tool, we applied a linear regression to Coaton’s tabulated (power, envelope temperature) data points. This linear trend is not intended to imply that the fundamental radiation physics is linear; rather, it serves as a convenient interpolation scheme between experimentally reported values within a narrow range of power (operating range). The actual radiative behavior is governed by the Stefan–Boltzmann law, which we apply explicitly to compute the net radiative flux.

For each lamp power setting in our experiments, the corresponding interpolated envelope temperature *T*_env_ is used to compute the outward radiative heat flux

$$q_{\mathrm{rad}}=\varepsilon \sigma (T_{\mathrm{env}}^{4}-T_{\mathrm{amb}}^{4})$$ 3.1

where ε is the effective emissivity of the quartz envelope and σ is the Stefan–Boltzmann constant. These *T*_env_ and *q*_rad_ values are then applied to the lamp-shaped void surfaces in our model as the lamp temperature and isotropic radiative flux boundary conditions, respectively. In our model, the “lamp temperature” therefore represents the outer-envelope radiating temperature, i.e., the temperature of the surface actually interfacing with the modeled volume of the reactor chamber.

To avoid ambiguity, we clarify the boundary conditions applied at 0% lamp power. In our model, 0% power corresponds to an envelope temperature of 526 K and a net radiative flux of zero. This choice is justified by combined effects of two important physical factors in the actual reactor system: (i) residual lamp warm-up heating and (ii) thermal equilibration with the preheated reactor environment. We provide a further explanation for these two factors below.

First, Coaton’s experimental data show that the lamp envelope exists at an elevated temperature above 500 K at the start of the operating power range (i.e., at nominally zero power).^46^ In fact, we also observe that at 0% power setting in the software controlling the IR heater, the lamps are on and the sample starts to heat up slightly. These observations result from the fact that in tungsten–halogen lamps, a minimum “warm-up” or “keep-warm” current typically flows after lamp activation. As a result, the lamp envelope remains significantly above ambient temperature, even when the filament is not emitting appreciable radiation.

Second, in our custom reactor, the IR chamber is directly adjacent to a resistive preheater, which brings the upstream hardware and surrounding gas to substantially above room temperature before the IR heating begins. Experimental measurements confirm that the IR chamber and its components, including the lamp envelopes, stabilize at temperatures well above ambient temperature due to conductive and radiative coupling with this preheater. Therefore, even if the lamps have not yet begun radiating, their quartz envelopes do not start at 298 K in practice.

For these reasons, assigning an envelope temperature of 526 K with zero radiative flux at the 0% power setting provides a physically realistic representation of the lamp boundary condition. The flux is correctly set to zero because no significant radiation is emitted at this state, while the elevated envelope temperature accurately reflects the real thermophysical conditions at the start of each simulation. This distinction between the two separate boundary conditions of envelope temperature (a wall-state variable) and radiative flux (a source term) allows our CFD model to capture the thermal inertia inherent in the quartz lamps while still enforcing correct radiative behavior.

In order to validate this approach described above, we ran multiple simulations at different lamp power values and found excellent agreement between the simulated and experimental thermocouple temperatures. A plot of the experimental (red) and simulation (blue) temperatures at 126 s is shown in Figure 9. As shown, the simulation results closely match the experimental data, and their fits are similar as well in both value and sublinear trend of the dependence of substrate temperature on the IR heater power setting. Hence, we demonstrate that by calibrating the lamp surface boundary condition of temperature and using the Stefan–Boltzmann law to compute radiative flux, our model captures both the magnitude and the dynamics of heating in our multizone RTP-CVD reactor.

**Figure 9:**
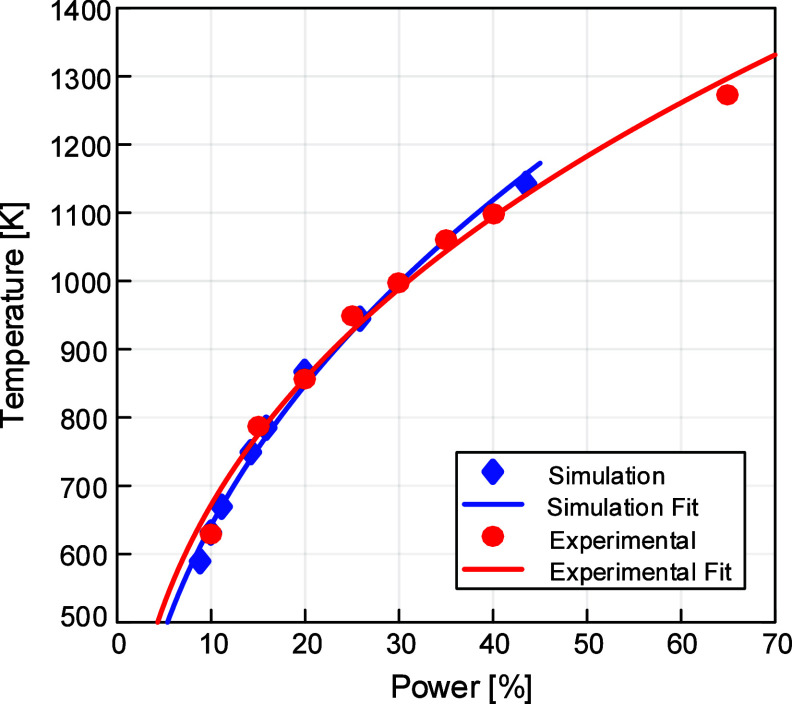
Comparison of experimental and simulation temperatures at 126 s for various powers, demonstrating that our simulation results are in good agreement with experimental results. Experimental measurements are obtained using the thermocouple located directly beneath the sample midpoint.

The agreement between the simulation and experimental temperatures was further enhanced through several minor tweaks. First, the initial fixed temperature of each domain was changed from 298 K (room temperature) to 328 K to better represent the preheater present in the reactor immediately upstream from the IR chamber. Second, the lamp flux value for each power was multiplied by a radiative efficiency term, obtained from Jenkins et al.,^34^ which provides a relation between radiative efficiency and lamp temperature. These values were adjusted for our purposes and ranged from 0.5 (50%) at 0% power to 0.9 (90%) at 100% power.

### 3.4 Thermocouple Spatial Uniformity Results

When we analyze the simulation results, we choose the top face of the catalyst chip, calculate an area average over this face, and plot temperature vs time. For the thermocouple, we use the same method at the end of the thermocouple, located inside the paddle just beneath the center of the chip, as shown in the thermocouple schematic in Figure 4. Additionally, we can demonstrate spatial uniformity in the thermocouple temperature results by plotting the thermocouple temperature along its length at several time points.

The spatial temperature distribution along the thermocouple length is evaluated not as an end in itself but as an important diagnostic of model fidelity. Because the thermocouple junction is embedded inside the quartz paddle and shielded from direct radiation, the temperature it registers depends on a balance of conduction along the thermocouple wires and conduction through the surrounding fused-silica paddle. A physically correct thermocouple model should therefore display minimal axial gradients near the junction, indicating that the measured temperature is truly representative of the local environment beneath the substrate. Large gradients or nonsmooth profiles would indicate inaccurate material properties or nonphysical thermal resistances. Evaluating the temperature profile along the thermocouple length therefore serves as an internal consistency check that the simulated thermocouple measurement reflects the physical reality.

Figure 10a illustrates this uniformity for 15% lamp power at the final time step of each of the simulation legs. The thermocouple contour plots in Figure 10b–e, also for 15% lamp power at the last time step of each of the simulation legs, further exemplify this spatial uniformity. In Figure 10b–e, the central warm region corresponds to the thermocouple body, while the cooler boundaries surrounding it represent the cavity inside the paddle. These contours, therefore, show both the thermocouple and its immediate thermal environment. The region appears at a lower temperature due to both lower thermal conductivity and the weak direct coupling to the substrate heating.

**Figure 10:**
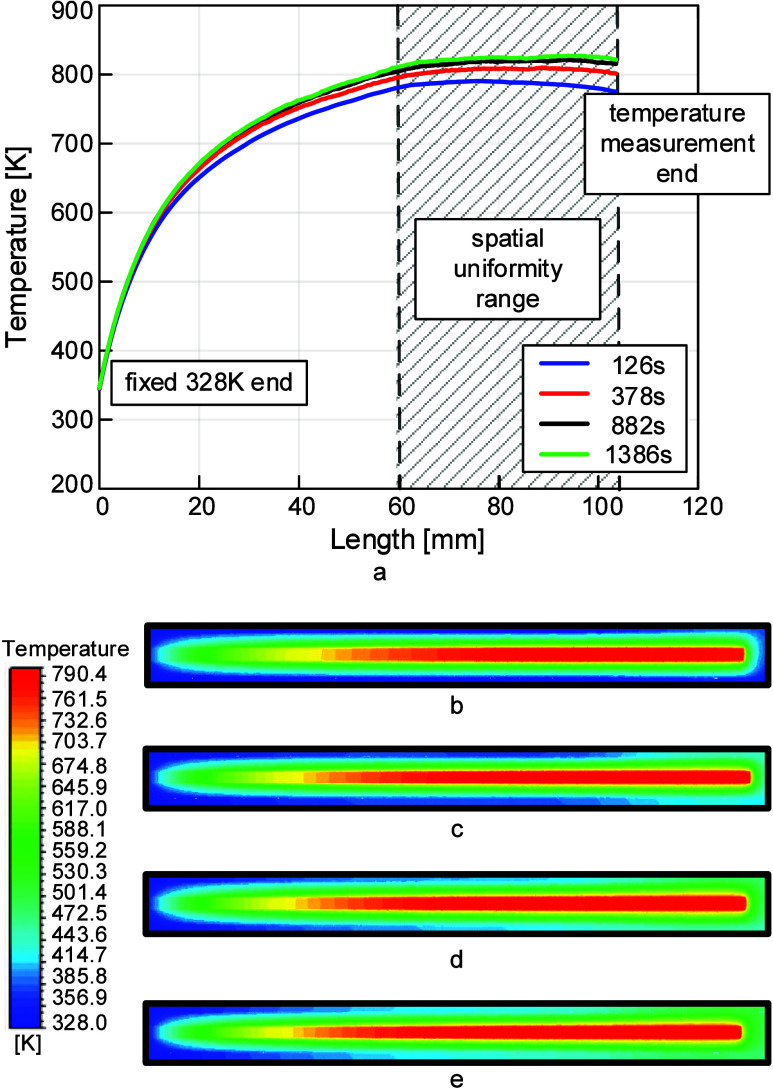
(a) Simulated temperature along the axial length of the thermocouple at 15% lamp power at the final time step of each of the simulation legs. This shows that the region near the junction attains a uniform temperature profile. This confirms that the thermocouple reading is representative of the local thermal environment beneath the substrate. (b−e) Cross-sectional temperature contours at the last time step of each of the simulation legs for 15% lamp power. The warm central region corresponds to the thermocouple body, while the cooler surrounding region corresponds to the cavity inside the quartz paddle. These plots therefore show both the thermocouple and the adjacent gas domain, illustrating the local thermal uniformity near the junction.

### 3.5 Stitching Time Stepping Scheme to Compare Catalyst Chip and Thermocouple Temperatures

Deciding on an appropriate time step that accurately captures simulation data while minimizing computation time is a critical part of any CFD project. A “stitching” approach was used to complete simulations for longer durations. This allowed us to check simulation results intermittently, and if any issues were present, we would be able to adjust the simulation early on without waiting days for completion. The first few seconds of our simulations experience the most dramatic rise in temperature; thus, it was hypothesized that 0–2 s should have a small time step of 0.0025 s. To “stitch” this first simulation to the next one, the final time step of this leg is used as the initial value for the next leg. The same stitching method is used for all following legs, where the last value of the previous leg is used as the initial value for the next leg. The time stepping scheme used for the next leg multiplied the time duration of the previous leg by 2 and multiplied the time step by 2 or 4. So, the second leg spanned 2 to 6 s with a time step of 0.01 s, the third leg 6 to 14 s with a time step of 0.04 s, and the fourth leg 14 to 30 s with a time step of 0.08 s. The final leg spanned 30 to 62 s with a time step of 0.16 s, which was the largest we were able to use without compromising results for the catalyst chip temperature. Thus, for all simulations that were run longer than 62 s, a time step of 0.16 s was used, if the chip temperature was the main focus. These time step values were determined through extensive simulations to identify the largest time step for each leg that could be used without affecting the chip temperature.

This study also revealed that while thermocouple temperature was independent of time step, the chip temperature was not. Figure 11 compares various time stepping schemes, showing that the thermocouple temperature does not change with time step size. A similar plot for the chip is provided in Supporting Information Section S7, showing that the temperature does change with time step size. Therefore, simulations focused solely on thermocouple temperature could use time steps larger than 0.16 s. As a result, these simulations could run for longer durations since they took less computational time. In general, when analyzing thermocouple temperature only and not chip temperature, both the time duration and time step were doubled for each leg. For example, 0 to 126 s had a time step of 0.16 s, 126 to 378 s had a time step of 0.32 s, and so on to the desired simulation time. This scheme was used for 10 and 5% lamp power up to a 0.64 s time step, beyond which Ansys CFX encountered issues. For 15% lamp power, 0.16 s was used for the first two legs.

**Figure 11:**
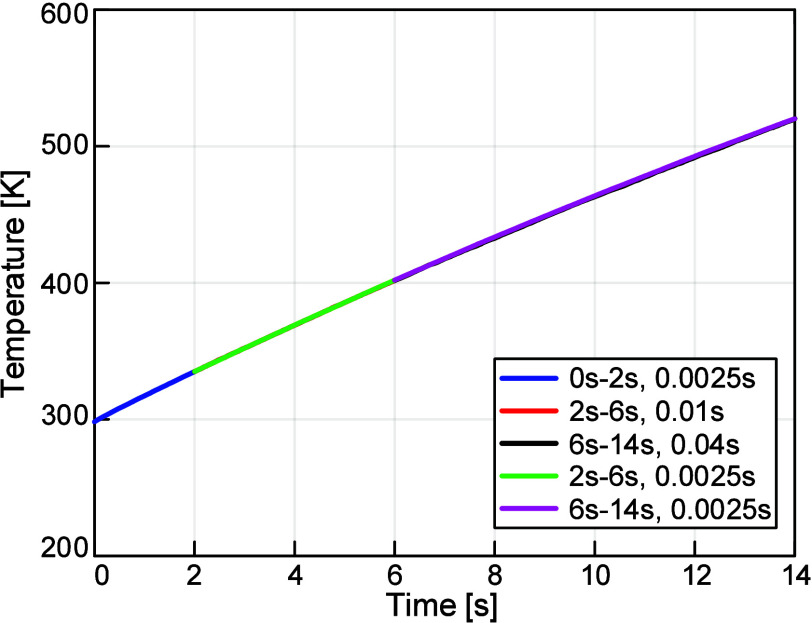
Results of thermocouple time step investigation, demonstrating that the thermocouple temperature is unaffected by the time step size.

### 3.6 Model Validation of Temperature Rise: Comparison with Experimental Results

The main method of validating our simulation results was to compare them with experimental results at the same power percentages. Since our reactor outputs temperature data read from the thermocouples present inside the IR chamber, it was decided that reading the simulation temperature from the end of the thermocouple would most accurately represent the experimental results. Figure 12 compares the simulated and experimental thermocouple temperatures at lamp power values of 5, 10, and 15%. Contour plots generated in Ansys CFD-Post are also provided for several points in time for 5 and 15% power. A zoomed-in temperature contour plot of the thermocouple and its surrounding air space is also shown.

**Figure 12:**
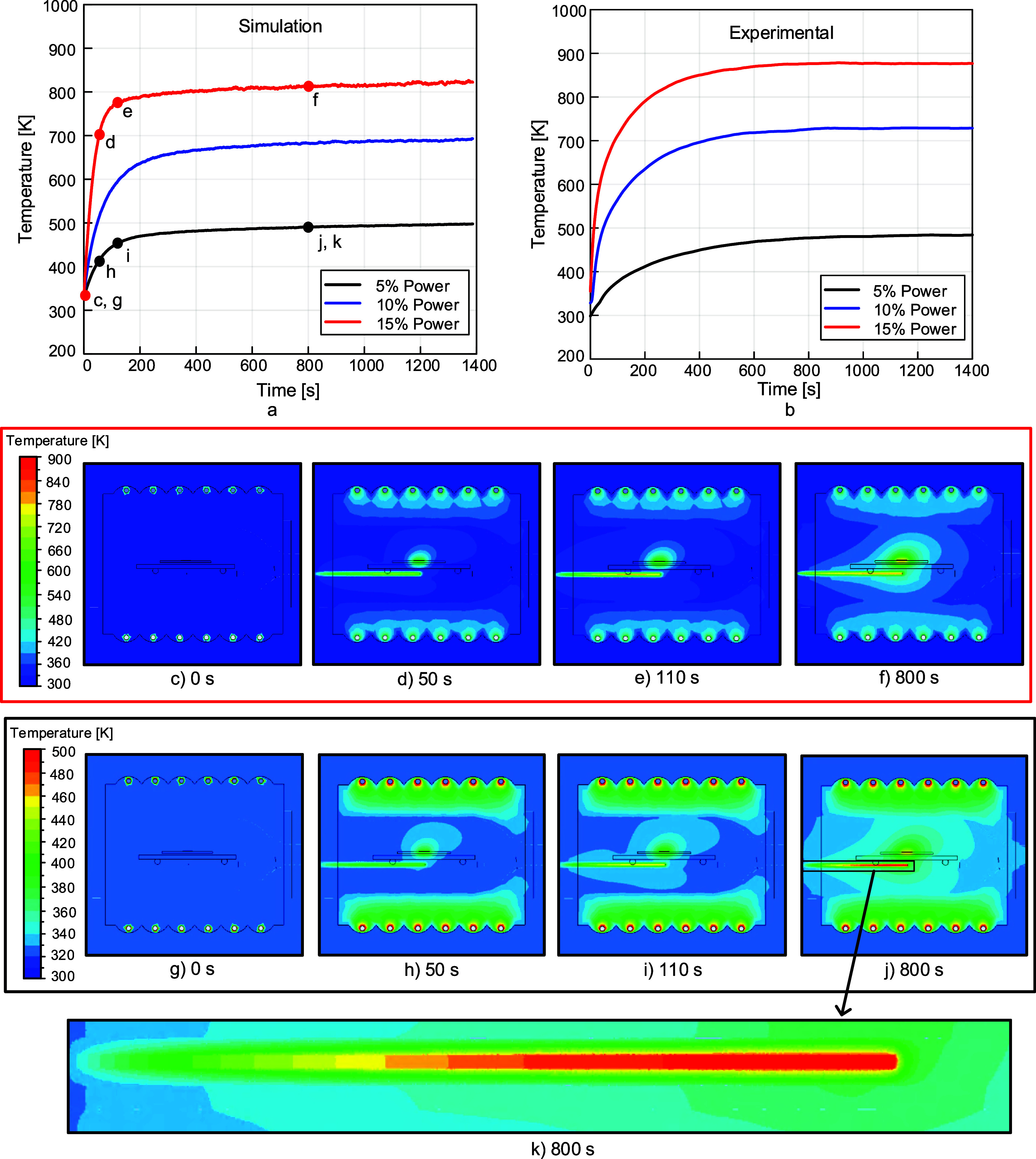
(a) Simulation thermocouple temperatures vs time and (b) experimental thermocouple temperatures vs time, demonstrating good agreement between simulation and experimental results for 5, 10, and 15% powers. The labels of the contour plots of the IR chamber shown in (c) through (f) and (g) through (k) correspond with the letter labels in (a), where (k) provides a more detailed look at the temperature distribution of the thermocouple for 800 s at 5% power. Images used courtesy of ANSYS, Inc.

As can be seen from the comparison in Figure 12, our simulations agree with the experimental results when it comes to the effect of IR heating power. Also, the steady-state temperatures reached in each case are in agreement with the experimentally measured temperatures using the thermocouple. Importantly, the temperature ramping kinetics are different with a much more smoothed curve in the experimental data. This highlights a big advantage of our simulations that can capture the true extremely high ramp rates of temperatures that are difficult to capture experimentally.

### 3.7 Spatiotemporal Evolution of Temperature across the Catalyst-Coated Chip

The catalyst chip temperatures from the same simulations are listed in Figure 13. Though time step independence for the chip has not yet been achieved, these simulations are still valuable. They allow us to examine the temperature distribution on the top side of the chip, as shown in the contour plots in Figure 13. The color bars (legends) use a small range of 3 K, as temperature uniformity is critical in CNT growth. This color bar range was kept constant across all contour plots for comparison, but contour plots with color bars scaled to each power percentage are provided in Supporting Information Section S8. The contour plots labeled e, f, and g in Figure 13 show the dependence of chip temperature profiles on lamp power, with much lower temperatures observed for 5% lamp power compared to 10 or 15%. It should also be mentioned that the catalyst chip is very small, 10 mm in width and length, and 0.525 mm thick. The temperature change across the chip (Delta T) is examined in more detail in the Supporting Information Section S8.

**Figure 13:**
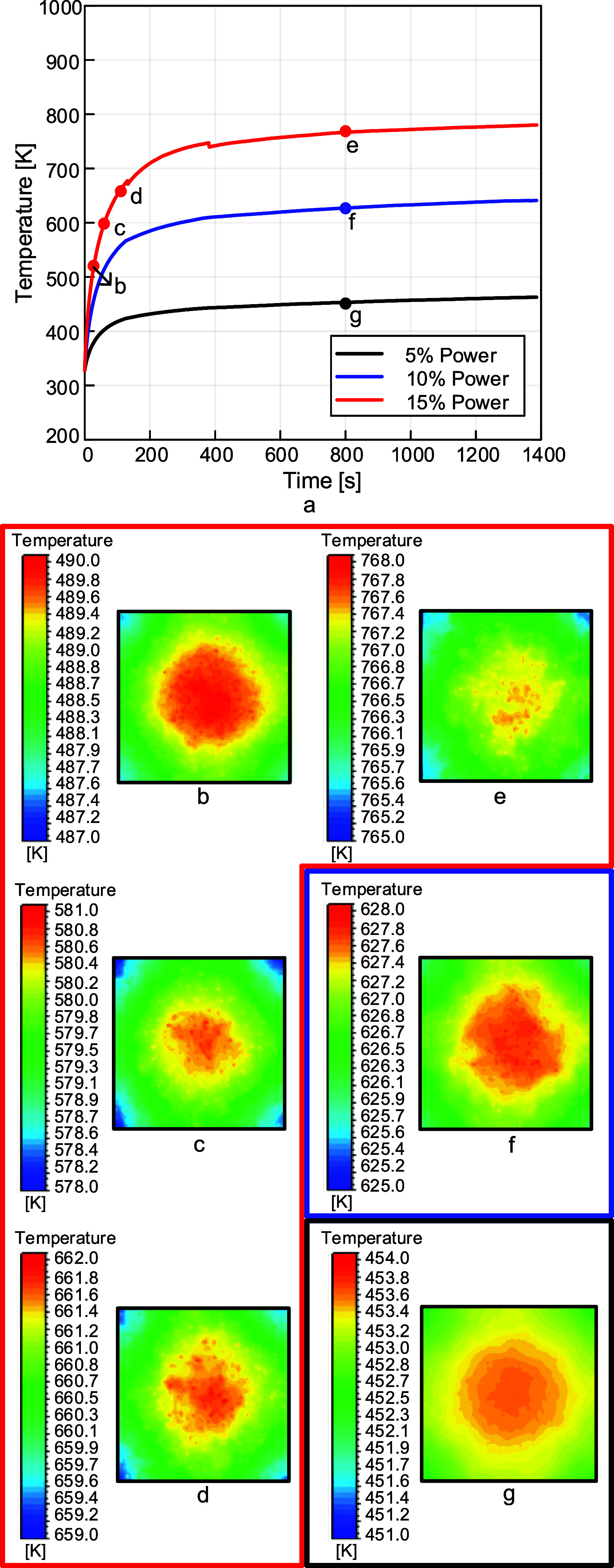
(a) Plot of the temperature on the top surface of the catalyst chip over time. (b–d) Contour plots for the top surface of the chip at 20, 50, and 110 s, respectively, for 15% power. (e–g) Contour plots at 800 s for 15, 10, and 5% power, respectively. The contour plot letter labels correspond with the letters in the chart shown in (a). Though we do not have time step independence for the catalyst chip, these plots are still useful for examining temperature nonuniformities across the chip.

Finally, we comment on the magnitude of the substrate temperature nonuniformity predicted by our model and how it compares to expectations from other reactor types. Direct experimental mapping of the 2D temperature field on the catalyst chip surface in an enclosed RTP-CVD reactor is extremely challenging, because common noncontact techniques (infrared cameras, multipoint pyrometry) are not easily implemented inside the quartz tube and are strongly affected by the evolving emissivity of the catalyst and growing CNT forest. For this reason, most prior IR-lamp-based RTP studies report only single-point or averaged temperature measurements rather than spatial temperature maps. In our case, we first validate the absolute temperature and transient response of the model against thermocouple data and then use the validated model to extract in-plane temperature variations Δ*T* across the 10 × 10 mm chip (Figures S6 and S7). Under the conditions studied here (5–15% lamp power), the Δ*T* values are a small fraction of the absolute substrate temperature. This level of nonuniformity is comparable to what is generally considered acceptable in rapid thermal processing tools and is consistent with the high geometric uniformity of millimeter-scale CNT forests observed in our previous multizone RTP-CVD experiments in the same reactor.^47^ Compared to conventional hot-wall tube furnaces, which offer good radial uniformity but limited dynamic control, our IR-lamp-based RTP system thus achieves both fast temperature ramping and small in-plane temperature nonuniformity on the substrate.

The results presented in this article provide new insights that have not been seen in previous works. For example, Logerais et al.^32^ present an RTP CVD model and shows a relation between lamp power and chip temperature but does not provide plots of the temperature distribution on the chip. As discussed previously, these spatiotemporal plots of temperature on the chip can provide information about nonuniformities during CNT growth, which enables explaining experimental results of geometric nonuniformity of CNT forests.^11^ Logerais et al.^32^ also mention the use of a thermocouple for temperature measurement but do not include it in the model. Hence, our more comprehensive modeling approach can be used to interpret experimental results, design substrate holders, and process recipes to achieve desired spatial maps of temperature over time.

### 3.8 Discussion of Heat Loss in This Model

To account for the small differences between the experimental and simulation temperatures, an investigation of the heat losses in the IR chamber was conducted. This was done in postprocessing, as Ansys CFX offers many thermal values that can be plotted for any domain from the simulation in Ansys CFD-Post, Release 2021 R2. In this case, the values considered were the wall heat flux, wall conductive heat flux, wall convective heat flux, wall absorbed radiation flux, and wall radiative heat flux. Each of these values was plotted over time for the fluid and solid side of the thermocouple, the fluid and solid side of the inner walls of the IR chamber, and the outer walls of the IR chamber, which only have a solid side. It was decided to conduct this investigation with 5% lamp power, but any of the three lamp powers discussed in the results could be used for the same purpose. This investigation was conducted only for the first leg of the simulation from zero to 126 s, because the most drastic heating occurs during this time frame.

A more detailed description of the results of this investigation is presented in Supporting Information Section S9. The conclusion of the investigation was that not taking into account convection from the cooling lines or fans on the outer walls of the reactor is the main reason for the temperature differences between our simulation and experimental results. This conclusion is supported by the plots shown in the Supporting Information, where it can be seen that conduction is the only mode of heat transfer on the outer walls in our model.

### 3.9 Model Scope and Path toward Full High-Temperature CVD Integration

The present work focuses on resolving the extreme thermal transients generated by rapid IR heating and on validating the radiative and conductive heat transfer pathways that dominate during this initial rapid heating regime. Some of our simulations are performed at modest lamp powers (5–15%) that keep the system below typical CNT growth temperatures so that the model can be validated directly against embedded thermocouple data without introducing confounding effects from gas-phase reactions or evolving catalyst optical properties.

#### Extending This Model to Capture High-Temperature CVD of Nanocarbons

This heating model is the foundation upon which more comprehensive high-temperature CVD simulations can be built. Future work can incorporate temperature-dependent spectral radiation within the fused-silica tube, gas-phase absorption and emission at elevated temperatures, precursor chemistry, and the evolving optical and thermal properties of CNT forests as they grow. These extensions require a validated thermal baseline, which is established in the present study. Therefore, the model presented here is a necessary and robust first step toward a full multiphysics simulation of CNT growth in rapid thermal CVD reactors.

#### Sensitivity of the Model to Thermal and Radiative Parameters

Although the present work does not involve chemical reaction kinetics or gas-phase decomposition pathways, several parameters strongly influence the thermal behavior of the RTP-CVD system. Accordingly, we performed extensive sensitivity analyses of the key thermal and radiative properties that govern the heating dynamics. These include (i) the sensitivity of thermocouple behavior to emissivity, heat capacity, density, and thermal conductivity; (ii) the sensitivity of lamp envelope temperature and radiative flux to different power-to-temperature calibration methods; and (iii) mesh refinement and time stepping sensitivity to ensure numerical independence in regions of steep thermal gradients. These analyses collectively identify the parameters that most strongly influence temperature evolution in the reactor and therefore can complement and support kinetic-parameter sensitivity in a chemical reaction model in the future. By establishing which thermal properties dominate system behavior, the model provides a robust foundation for the future integration of gas-phase reaction kinetics and high-temperature CVD processes.

### 3.10 Implications beyond Our Specific Reactor Design

Although this study focuses on a custom-designed multizone infrared rapid thermal CVD reactor for carbon nanotube synthesis, the modeling framework developed here is broadly extendable to a wide range of radiatively or rapidly heated reactor architectures. Several components of our FEM methodology map directly onto other systems used in semiconductor processing, thin-film synthesis, catalysis, and nanomaterial growth.

#### Radiative Boundary Condition Methodology Applicable to Any Lamp-Heated Reactor

The core strategy used here, deriving lamp envelope temperatures from experimental or manufacturer data, interpolating within the operational range, and computing net radiative flux using the Stefan–Boltzmann law, is fully generalizable. Analogous workflows can be applied to lamp-heated RTP systems used in semiconductor processing,^48^ transparent-window cold-wall CVD tools,^49^ lamp-heated atomic layer deposition (ALD) reactors,^50^ and lamp-based annealing or phase-transformation systems.^51^ Many radiatively heated reactors rely on empirical correlations between the electrical power and temperature. The simulation method demonstrated here provides a physically grounded strategy for converting power settings to accurate thermal boundary conditions.

#### Monte Carlo Radiation Modeling in Lamp-Dense Geometries

The Monte Carlo radiation modeling approach is particularly relevant to reactors with multilamp arrays.^52^ These can include reflective housings and complex optical interactions that further necessitate a comprehensive FEM model like the one we developed in this work. Moreover, as lamp-based thermal processing continues to shrink spatially and increase dynamic control, radiation-transport modeling becomes essential for predicting local heating rates and in-plane temperature gradients. The methodology presented here is thus broadly transferable.

#### Model Validation Strategy Generalizable to Closed or Emissivity-Changing Reactors

Our validation strategy, combining a physically accurate composite-material thermocouple model, envelope-temperature-derived lamp boundary conditions, and experimental transient thermocouple data is particularly valuable for reactor systems where direct, spatially resolved temperature measurements are impossible. This includes enclosed RTP reactors with quartz tubes and CVD systems where emissivity changes during growth (graphene, CNTs, and Si nanowires), as well as lamp-heated ALD systems with opaque or reflective internals. Hence, this type of model-validating approach fills a gap in radiatively heated, closed-geometry reactors, where optical pyrometry is unreliable or obstructed.

In summary, although our physical reactor geometry is unique, the conceptual and methodological contributions of this work, radiative-power calibration, Monte Carlo radiation modeling, material property sensitivity analysis, and validated transient multizone FEM simulation address fundamental challenges common to radiatively driven thermal processing. These modeling tools can therefore be readily applied to optimize other lamp-based CVD, ALD, RTP, and thin-film processing systems used throughout materials science and semiconductor manufacturing.

## 4 Conclusions

In this article, we presented a CFD model that captures the extreme rapid thermal dynamics and its spatial distributions within the IR chamber of a custom CVD reactor used for the catalytic growth of carbon nanotubes. We provided a description of our custom-designed system and reviewed the challenges in creating extreme reactors for process control, particularly without the ability to precisely characterize the spatiotemporal temperature evolution. Hence, our work presented here on modeling such reactors is fundamental, as it provides a unique tool not only for understanding the thermochemical processes but also for design purposes toward unprecedented process control. It was determined that the best radiation model for our case was Monte Carlo, with a transfer mode of surface to surface or participating media depending on the domain. Simplifications from the actual reactor and the full model of the geometry were explored. Modeling the thermocouple accurately presented many challenges, such as developing the composite model. Modeling the IR lamps required several tests to determine the best geometry, mesh density, and boundary conditions. A methodology was developed to model IR lamp heating by relating lamp power to two physics-grounded boundary conditions: envelope temperature and radiative flux. We demonstrated time step- and mesh-independent results for thermocouple temperature and spatial uniformity in those temperature results. The model showed good agreement with experimental temperature data at power percentages of 5, 10, and 15%. An investigation of the heat losses was also performed, leading to more insight into the temperature nonuniformities present on the catalyst chip. It was determined from this investigation that not taking into account the cooling on the outside walls of the IR chamber was one of the main contributing factors to the difference between experimental and simulation temperatures. Overall, our model has proven useful in exploring temperature nonuniformities that can lead to nonuniform CNT growth.

In the future, this model can be used for a range of simulation studies. The first task will be to achieve time-step-independent temperature data for the catalyst chip. The material of the chip and the support wafer can be varied, and the fluid that is injected into the process tube can be changed from helium to argon to investigate the effects on catalyst formation. As mentioned previously, uniformity of the temperature on the surface of the catalyst chip is essential. This can be further investigated by changing the boundary conditions at the edges of the chip and changing the tray and support wafer design. The effect that the CNTs have on the catalyst chip temperature can be explored by adding CNTs to the model on top of the chip, as their high absorptivity may affect the temperature of the catalyst beneath them. As mentioned previously, the cooling lines and fans present on the outside of the IR chamber can be added to the model to further investigate the effects of convective cooling on the temperature uniformity. Components of the process tube that were neglected, such as the thermal baffle and helical injector, can also be added to evaluate their effects.

## Supplementary Material



## Notes

Description of experimental temperature measurements; material properties used in the model; overview of radiation models available in Ansys CFX; schematic of tray, wafer, and paddle configuration; complete domain and interface settings used in Ansys CFX; results of thermocouple material property investigations; investigation of catalyst chip temperature dependence on time step size; analysis of spatial temperature nonuniformities (Delta T) across the chip surface; and heat loss investigation for different chamber surfaces and interfaces (PDF)
