# Supplementary material for: Modeling Extreme Rapid Thermal Processing in Chemical Vapor Deposition Reactors for Catalytic Growth of Carbon Nanotubes
Source: Ind Eng Chem Res. 2025 Dec 15;64(51):24621–37. doi: 10.1021/acs.iecr.5c04214 (PMC12750536; doi:10.1021/acs.iecr.5c04214)
Supplement: Supplementary file 1 [file ie5c04214_si_001.pdf]

Supporting Information for

Modeling Extreme Rapid Thermal Processing in Chemical Vapor Deposition Reactors for Catalytic Growth of Carbon Nanotubes

Julia Aurenzi[1]*, Moataz Abdulhafez*[2]*, Golnaz Najaf Tomaraei*[2]*, Shervin Sammak*[1]*, Mostafa Bedewy*[1,2,4*]

[1] Department of Mechanical Engineering and Materials Science, University of Pittsburgh, 3700 O'Hara Street, Pittsburgh, PA 15261, USA

[2] Department of Industrial Engineering, University of Pittsburgh, Pittsburgh, PA 15261, USA

[3] Department of Chemical and Petroleum Engineering, University of Pittsburgh, Pittsburgh, PA 15261, USA

Corresponding Author

*Mostafa Bedewy (mbedewy@pitt.edu)

S1. Description of experiments

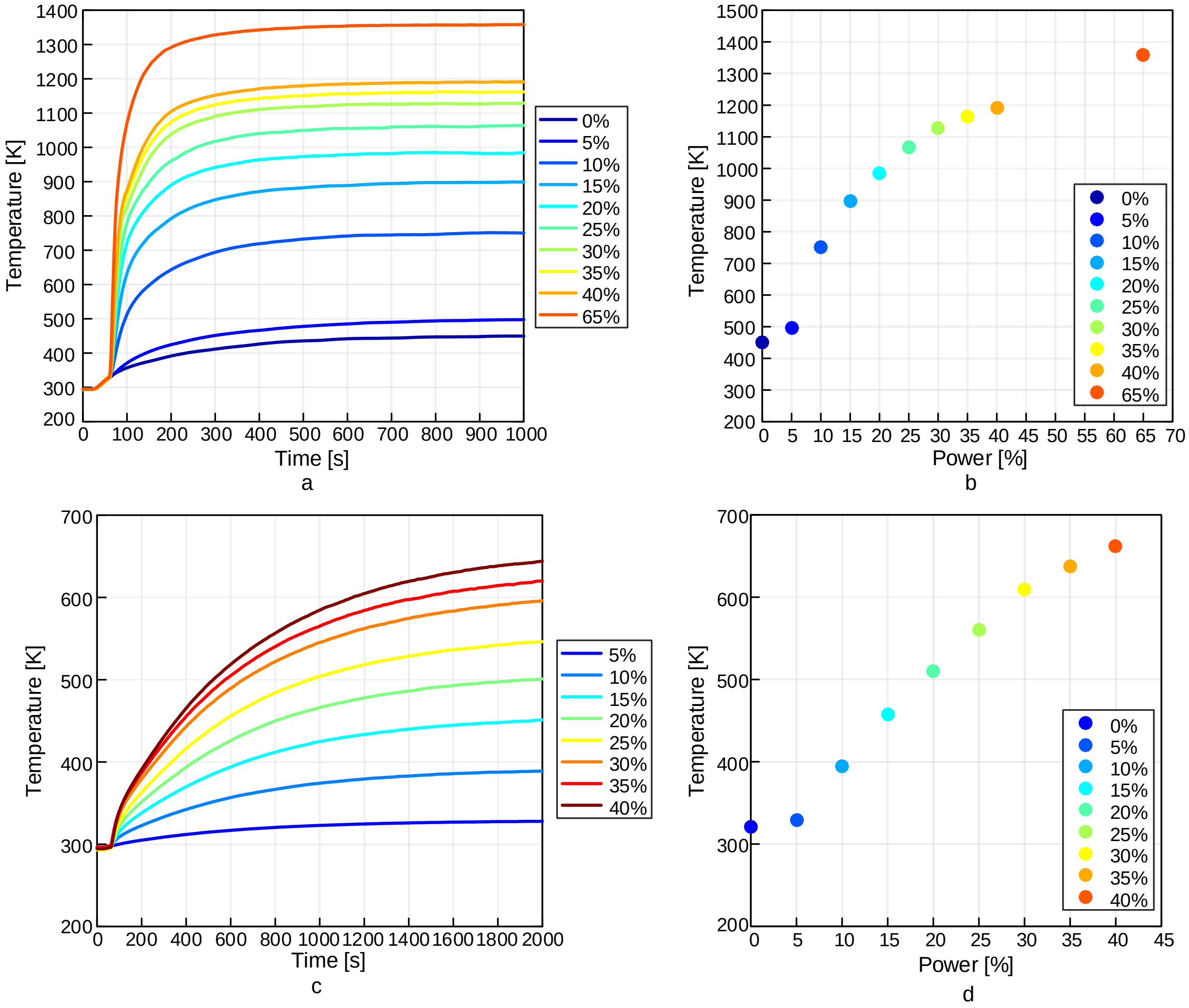

Figure S1. Temperatures for each power percentage over time for IR heater (a) center zone and (c) preheater, and steady state temperatures for various power percentages of (b) center zone and (d) preheater.

S2. Material properties

The table below provides a detailed description of the material properties for each material used in the model.

Table S1. Material Properties used in the model.

Material	Fused Silica	Gold	Silicon	Air at STP	He at STP	TC
State	solid	solid	solid	gas	gas	solid
Molar Mass	60.08 g/mol	196.97 kg/kmol	28.0855 kg/kmol	28.96 kg/kmol	4.00 kg/kmol	51.378136 kg/kmol
Density	2220 kg/m^3	19300 kg/m^3	2330 kg/m^3	1.284 kg/m^3	0.179 kg/m^3	6576.112416 kg/m^3
Specific Heat Capacity	745 J/kgK	129 J/kgK	712 J/kgK	1.0038e+3 J/kgK, constant pressure	5240 J/kgK, constant pressure	618.9632 J/kgK
Reference State	Temp: 300 K	Temp: 300 K	Temp: 300 K	Temp: 0C, Pressure: 1 atm	Temp: 0C, Pressure: 1 atm	Temp: 25 C
Reference Specific Enthalpy	0 J/kg	0 J/kg	0 J/kg	0 J/kg	-1.2982658e+5 J/kg	0 J/kg
Reference Specific Entropy	0 J/kgK	0 J/kgK	0 J/kgK	0 J/kgK	3.1034571e+4 J/kg/K	0 J/kgK
Thermal Conductivity	1.38 W/mK	317 W/mK	148 W/mK	2.428e-2 W/mK	1415e-4 W/mK	8.66936 W/mK
Dynamic Viscosity	n/a	n/a	n/a	1.725e-5 kg/ms	18.6e-6 kg/ms	n/a
Refractive Index	1.4585	1.16901	3.9766	1	1	1
Absorption Coefficient	0.1 1/m	7.0051e+7 1/m	646050 1/m	0.01 1/m	1 1/m	1 1/m
Scattering Coefficient	0 1/m	0.5 1/m	0 1/m	0 1/m	0 1/m	0 1/m
Thermal Expansivity	n/a	n/a	n/a	0.00366 1/K	0.00366 1/K	n/a

S3. Radiation models in Ansys CFX

The main objective of radiation modeling in Ansys CFX is to solve the radiation transport equation and obtain the source term for the energy equation (2.4) described previously. The spectral radiative transfer equation (RTE), according to the Ansys CFX-Solver Theory Guide[1] is shown in (2.6).

$$\frac{dI_v(\boldsymbol{r},\boldsymbol{s})}{ds} = \left(-(K_{av} + K_{sv})I_v(\boldsymbol{r},\boldsymbol{s}) + K_{\boldsymbol{av}}I_b(v,T) + \frac{K_{sv}}{4\pi}\int_{4\pi} dI_v(\boldsymbol{r},\boldsymbol{s}')\Phi(\boldsymbol{s}\cdot\boldsymbol{s}')d\Omega' + S\right) \qquad (2.6)$$

Where I_v is spectral radiation intensity which depends on position r and direction s, $\boldsymbol{r}$ is the position vector, $\boldsymbol{s}$ is the direction vector, s is path length, v is frequency, K_a is the absorption coefficient, K_s is the scattering coefficient, I_b is Blackbody emission intensity, T is local absolute temperature, Φ is the in-scattering phase function, Ω is the solid angle, and S is the radiaiton energy source term or particle-radiation interactions.

Solving the RTE is very time consuming, thus Ansys CFX includes directional and spectral approximation models. The radiation models involving directional approximations are Rosseland, P1, Discrete Transfer, and Monte Carlo. Each of these models also contain spectral approximations, or spectral models.

The Rosseland model simplifies the RTE for optically thick media by introducing a new diffusion term which contains a strongly temperature dependent diffusion coefficient.[1] This model is typically recommended for optical thicknesses greater than five. It is also important to note that this approximation is not valid near walls, because this model assumes that radiant energy emitted from other areas in the domain do not have influence on the local transport because they are quickly absorbed.[2] Thus, a boundary condition must be specified for wall treatment.

The P1 model simplifies the RTE by assuming that the radiation intensity is isotropic or direction independent at a given location in space.[1] At the walls, it is assumed that the radiation intensity coming into or going out of a wall are directionally independent, and a specific boundary condition is needed. The P1 model is typically recommended when the optical thickness is greater than one. When using this model, the diffuse fraction setting on walls is ignored, as this model only allows for diffuse opaque walls. It also treats all open boundaries, such as inlets, as fully transparent meaning that they absorb all outgoing energy and incoming energy is calculated as a blackbody.[2]

The Discrete Transfer model simplifies the RTE by assuming that the scattering is isotropic. This model is based on the discretization of the transfer equation along rays, and tracing the domain using multiple arrays leaving from boundary surfaces.[2] This model is only valid for optically thin or transparent media.

Lastly, the Monte Carlo model assumes that the intensity is proportional to the differential angular flux of the photons, where one can consider the radiation field as a photon gas, and then the absorption coefficient is the probability per unit length that a photon is absorbed at a given frequency.[1] Similar to the way Discrete Transfer model traces rays, this model tracks photons through a domain. As mentioned previously, it is also important to select an appropriate number

of histories when using the Monte Carlo model. This number of histories is used in the Monte Carlo statistics calculations[1] and is divided into several groups. Then, histories are selected from each group and their physical interactions are tracked through the domain. Some of the physical interactions include emission, absorption, and reflection. Once the calculation is complete, each of the groups divided from the histories provides values for the quantities of interest, and then the mean and standard deviation of each are calculated from the groups. Finally, a normalized standard deviation is calculated.

S4. Tray configuration

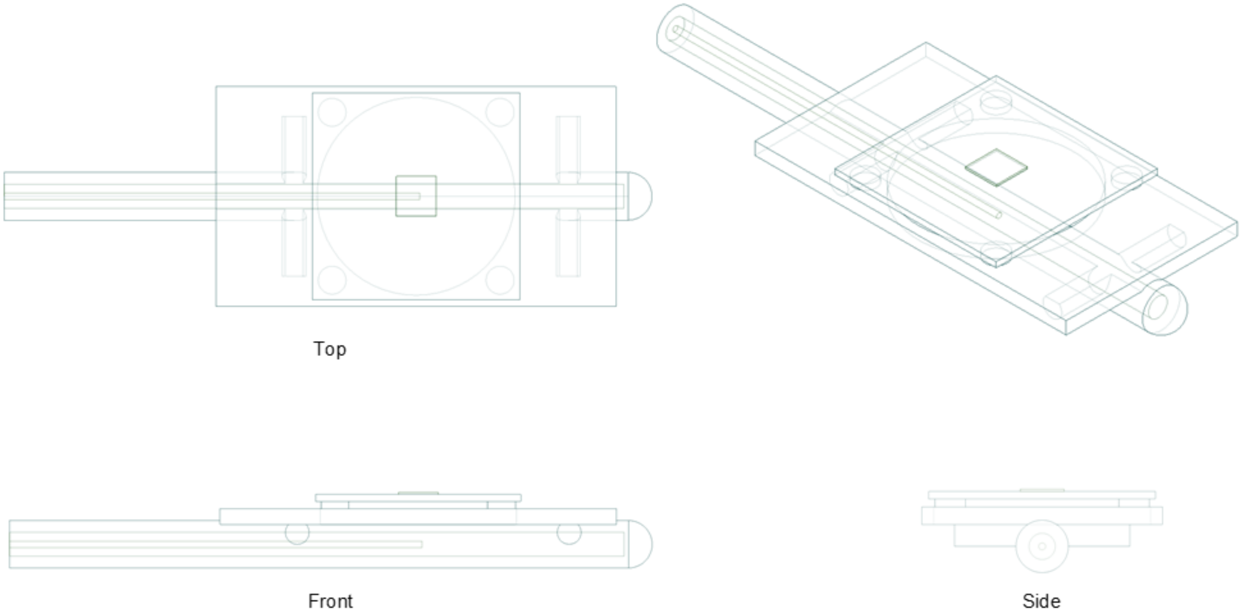

Figure S2. Details of tray, catalyst chip, and paddle configuration generated using SpaceClaim.

S5. Domain and interface settings

The following tables provide detailed descriptions of the settings used in Ansys CFX to define the domains and interfaces present in the model.

Table S2. Domain settings.

	Domain						
Label	Name	Type	Material	Heat Transfer	Thermal Radiation	Turbulence Model	First Leg Initialization
a	IR Chamber Walls	Solid Domain, Continuous Solid Morphology, Stationary Domain Motion, No Mesh Deformation	Gold	Thermal Energy	Monte Carlo, Participating Media, 1000000 Histories, Gray Spectral Model, No Scattering Model	N/A	328 K Temperature, Automatic Radiation Intensity
b	IR Chamber Fluid Boundary						
c	IR Chamber Fluid	Fluid Domain, Continuous Fluid Morphology, 1 atm Reference Pressure, Non Buoyant, Stationary Domain Motion, No Mesh Deformation	Air at STP	Thermal Energy	Monte Carlo, Surface to Surface, 1000000 Histories, Gray Spectral Model, No Scattering Model	SST, Automatic Wall Function	0 U,V,W Velocity, 1 atm Static/Relative Pressure, 328 K Temperature, Medium Turbulence (5% Intensity), Automatic Radiation Intensity
d	Reactor Process Tube	Solid Domain, Continuous Solid Morphology, Stationary Domain Motion, No Mesh Deformation	Fused Silica	Thermal Energy	Monte Carlo, Surface to Surface, 1000000 Histories, Gray Spectral Model, No Scattering Model	N/A	328 K Temperature, Automatic Radiation Intensity

Table S2. Continued.

e	Reactor Process Tube Fluid	Fluid Domain, Continuous Fluid Morphology, 1 atm Reference Pressure, Non Buoyant, Stationary Domain Motion, No Mesh Deformation	Helium at STP	Thermal Energy	Monte Carlo, Surface to Surface, 1000000 Histories, Gray Spectral Model, No Scattering Model	SST, Automatic Wall Function	0 U,V, Velocity, 0.0062 m/s W Velocity, 1 atm Static/Relative Pressure, 328 K Temperature, Medium Turbulence (5% Intensity), Automatic Radiation Intensity
f	Catalyst-coated silicon chip	Solid Domain, Continuous Solid Morphology, Stationary Domain Motion, No Mesh Deformation	Silicon	Thermal Energy	Monte Carlo, Participating Media, 1000000 Histories, Gray Spectral Model, No Scattering Model	N/A	328 K Temperature, Automatic Radiation Intensity
g	Paddle	Solid Domain, Continuous Solid Morphology, Stationary Domain Motion, No Mesh Deformation	Fused Silica	Thermal Energy	Monte Carlo, Surface to Surface, 1000000 Histories, Gray Spectral Model, No Scattering Model	N/A	328 K Temperature, Automatic Radiation Intensity
h	Paddle Inner Fluid	Fluid Domain, Continuous Fluid Morphology, 1 atm Reference Pressure, Non Buoyant, Stationary Domain Motion, No Mesh Deformation	Air at STP	Thermal Energy	Monte Carlo, Surface to Surface, 1000000 Histories, Gray Spectral Model, No Scattering Model	SST, Automatic Wall Function	0 U,V,W Velocity, 1 atm Static/Relative Pressure, 328 K Temperature, Medium Turbulence (5% Intensity), Automatic Radiation Intensity

Table S2. Continued.

i	TC	Solid Domain, Continuous Solid Morphology, Stationary Domain Motion, No Mesh Deformation	TC	Thermal Energy	Monte Carlo, Participating Media, 1000000 Histories, Gray Spectral Model, No Scattering Model	N/A	328 K Temperature, Automatic Radiation Intensity

Table S3. Domain boundary settings.

	Boundaries							
Label	Domain Name	Location	Type	Mass and Momentum	Wall Roughness	Heat Transfer	Thermal Radiation	Sources
a	IR Chamber Walls	Bottom<br>Top<br>Front Side<br>Back Side<br>Inlet Side<br>Outlet Side	Wall	N/A	N/A	Fixed Temperature, 328 K	Opaque, 0.02 Emissivity, 0 Diffuse Fraction	N/A
b	IR Lamps							
c	IR Chamber Fluid	IR Lamp Surfaces	Wall	No Slip Wall	Smooth Wall	Temperature, Based on Lamp Power	Opaque, 1 Emissivity, 1 Diffuse Fraction	Radiation Source, Isotropic Radiation Flux, Based on Lamp Power
		Wall Ends	Wall	No Slip Wall	Smooth Wall	Temperature, 328 K	Opaque, 0.02 Emissivity, 0 Diffuse Fraction	N/A
d	Reactor Process Tube	Wall Ends	Wall	N/A	N/A	Temperature, 328 K	Opaque, 0.02 Emissivity, 0 Diffuse Fraction	N/A
e	Reactor Process Tube Fluid	Inlet	Inlet, Subsonic Flow Regime, Medium Turbulence (5% Intensity)	Normal Speed, 0.0062 m/s	N/A	Static Temperature, 328 K	Local Temperature	N/A
		Outlet	Outlet, Subsonic Flow Regime	Static Pressure, 1 atm	N/A	N/A	Local Temperature	N/A
f	Catalyst-coated silicon chip	N/A						
g	Paddle	Wall End	Wall	N/A	N/A	Fixed Temperature, 328 K	Opaque, 0.02 Emissivity, 0 Diffuse Fraction	N/A
h	Paddle Inner Fluid	Wall End	Wall	No Slip Wall	Smooth Wall	Fixed Temperature, 328 K	Opaque, 0.02 Emissivity, 0 Diffuse Fraction	N/A
i	TC	Wall End	Wall	N/A	N/A	Fixed Temperature, 328 K	Opaque, 0.02 Emissivity, 0 Diffuse Fraction	N/A

Table S4. Interface settings.

Interface					
Label	Name	Type	Heat Transfer	Thermal Radiation	Mesh Connection
a,c	Default Fluid Solid Interface	Fluid Solid	Conservative Interface Flux, No Interface Model	Opaque, 0.02 Emissivity, 0 Diffuse Fraction	GGI (General Grid Interface)
c,d	OutsideTube_insideBox	Fluid Solid	Conservative Interface Flux, No Interface Model	Conservative Interface Flux	Automatic
d,e	insideTubeSolid_insideTubeFluid	Fluid Solid	Conservative Interface Flux, No Interface Model	Conservative Interface Flux	Automatic
e,f	Catalyst_insideTube	Fluid Solid	Conservative Interface Flux, No Interface Model	Opaque, 0.9 Emissivity, 1 Diffuse Fraction	Automatic
e,g	Paddle_insideTube	Fluid Solid	Conservative Interface Flux, No Interface Model	Conservative Interface Flux	Automatic
f,g	Paddle_Catalyst	Solid Solid	Conservative Interface Flux, No Interface Model	Conservative Interface Flux	Automatic
g,h	Paddle_insidePaddle	Fluid Solid	Conservative Interface Flux, No Interface Model	Conservative Interface Flux	Automatic
h,i	TC_insidePaddle	Fluid Solid	Conservative Interface Flux, No Interface Model	Opaque, 0.46 Emissivity, 1 Diffuse Fraction	Automatic

S6. Thermocouple material property investigations

Figure S3 below shows the results of the thermocouple density investigation. It can be seen that changing the density value did not change thermocouple temperature by much.

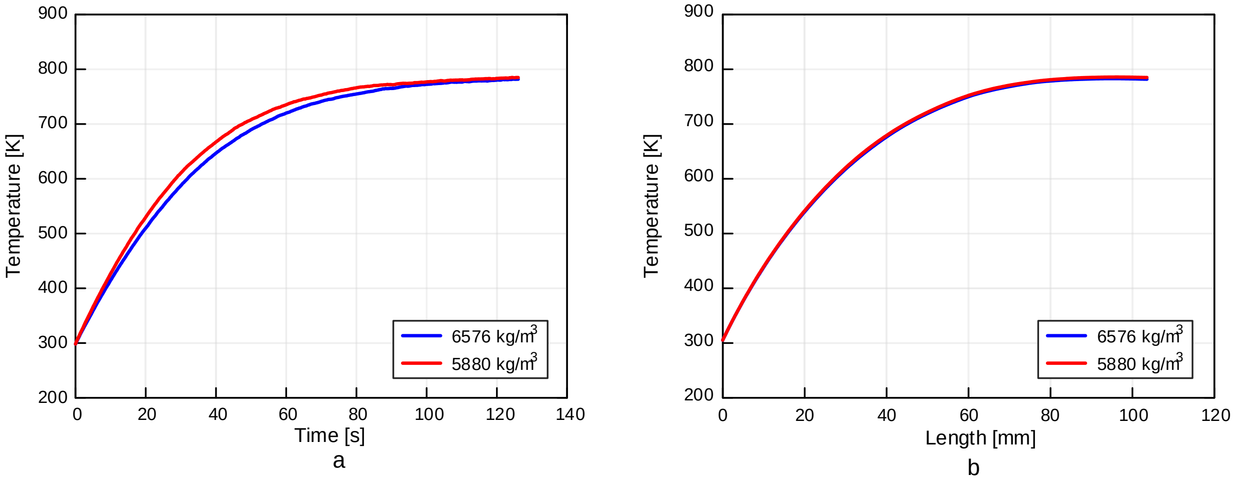

Figure S3. Results of thermocouple density investigation, (a) temperature vs time and (b) temperature vs length. The results demonstrate that changing the density of the thermocouple material does not have much of an effect on the temperature values.

Figure S4 below shows the results of the thermocouple specific heat investigation. Again, changing these values did not change the thermocouple temperature.

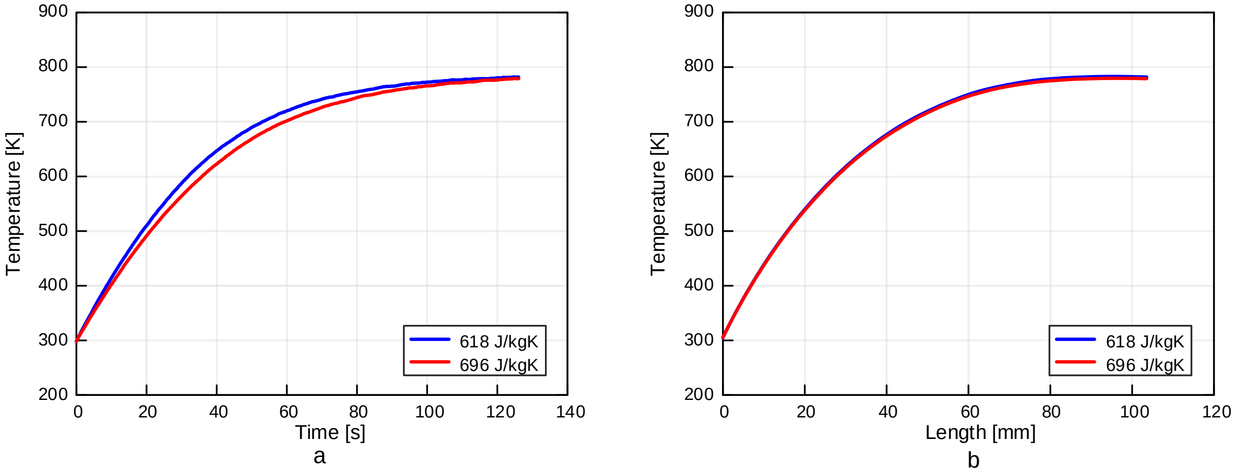

Figure S4. Results of the thermocouple specific heat investigation, (a) temperature vs time and (b) temperature vs length. The results demonstrate that changing the specific heat of the thermocouple material does not have a large effect on the temperature values.

S7. Catalyst-coated Si chip time step investigation

Figure S5 shows the results of the time step investigation for the catalyst chip, demonstrating that chip temperature is dependent on time step size, unlike the thermocouple.

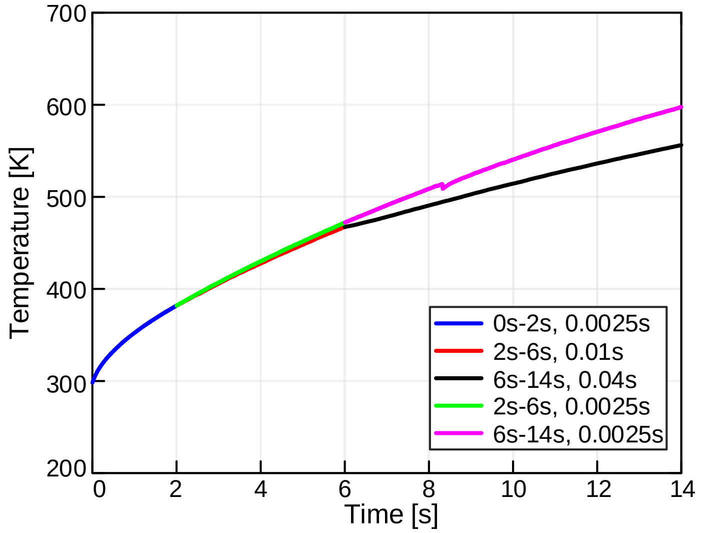

Figure S5. Results of catalyst chip time step investigation, demonstrating that the chip temperature is dependent on time step size.

S8. Catalyst chip Delta T investigation

Figure S6 provides an alternate version to **Figure 13,** where the color bars (legends) of the catalyst chip contour plots are adjusted for each simulation.

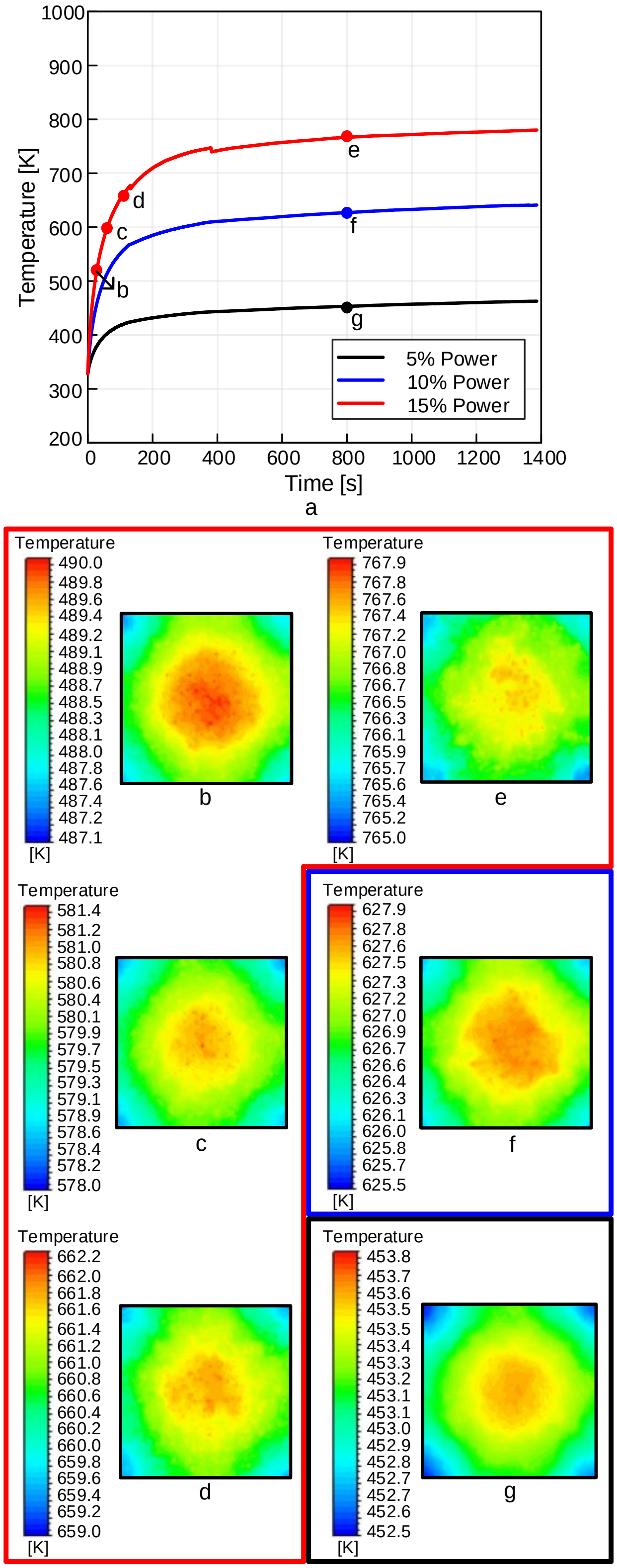

Figure S6. Alternate version of Figure 13, in which the temperature ranges of the heat maps are slightly different.

The plot in **Figure S7** gives more insight into the temperature nonuniformities on the surface of the chip shown in the contour plots in **Figure S6.Error! Reference source not found.** Here, temperatures at the edge of the chip and at the middle of the chip at 5%, 10%, and 15% lamp power for several points in time were obtained. The temperature difference across the chip is calculated using an average of the four corner temperatures, and an average of five points in the middle of the chip. These averages are then subtracted, creating a temperature difference, or Delta T as labeled on the plot, across the chip. This shows that not only is there temperature nonuniformity at earlier times in the simulation, but also at steady state; further exemplifying the nonuniformities shown in the contour plots of **Figure 13**. This is likely due to the fact that a cold-wall reactor is used, so the edges of the chips have lower temperatures due to the cooling that is occurring on the outer walls of the reactor. It can also be observed that the temperature difference increases as the power percentage increases. The implications of these temperature nonuniformities on the chip surface include catalyst nonuniformity and nonuniformity in CNT growth. Hence, the effect of this temperature distribution on CNT growth need to be explored in the future.

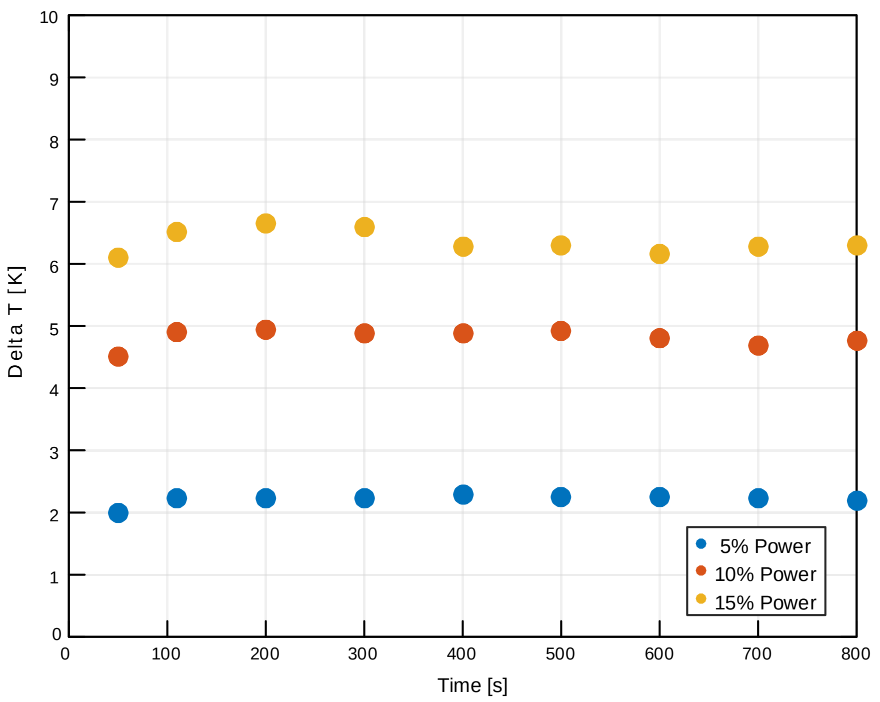

Figure S7. Temperatures on the top surface of the catalyst chip at several points in time for each power percentage, supporting the conclusion that temperature nonuniformities exist even at steady state.

S9. Heat loss investigation

As mentioned previously, a heat loss investigation was conducted to account for some of the differences between the experimental and simulation temperatures, an investigation of the heat losses in the IR chamber. The values examined were wall heat flux, wall conductive heat flux, wall convective heat flux, wall absorbed radiation flux, and wall radiative heat flux. Wall heat flux refers to the total heat flux into the specified domain, which includes the wall conductive and wall convective heat flux values.[2] Wall conductive heat flux and wall convective heat flux refer to the heat flux from conduction and convection heat transfer respectively. Wall absorbed radiation flux represents the absorbed heat flux due to radiation. Wall radiative heat flux represents the net radiative energy flux leaving a specified boundary. It is computed as the difference between the radiative emission and the incoming (absorbed) radiative flux. Though not included in Ansys, the wall emitted radiation flux was also considered and obtained by subtracting the wall absorbed radiation flux values from the wall radiative heat flux values. An area integration method was used, thus outputting values in watts (W) instead of W/m^2, and therefore the values will be referred to as heat instead of heat flux going forward.

Figure S8 shows heat loss investigation results for outer walls of the IR chamber. These plots show that the only mode of heat transfer on the outer walls is conduction, and that the heat losses eventually reach a steady state. There is no convection present on the outer walls because the model did not include the cooling lines or fans present in the actual reactor in order to simplify the CFD model. Not including the convective cooling on the outer walls in the model could possibly delay the steady state and may be why the model overestimates the final thermocouple and catalyst chip temperatures and underestimates the heating rates for the thermocouple and catalyst chip. Despite what the plot appears to present, the wall radiative heat is zero. This is known because the boundary conditions set on the outer walls of the reactor represent a mirror with an emissivity of 0.02 and a diffuse fraction of zero. Examining the plot on the left, it can be concluded that the reason for the noise giving the wall radiative heat a value other than zero is the noise from calculating wall absorbed and wall emitted heat. There is also the factor of numerical error which is a factor in any CFD model, especially one performing complex radiation calculations.

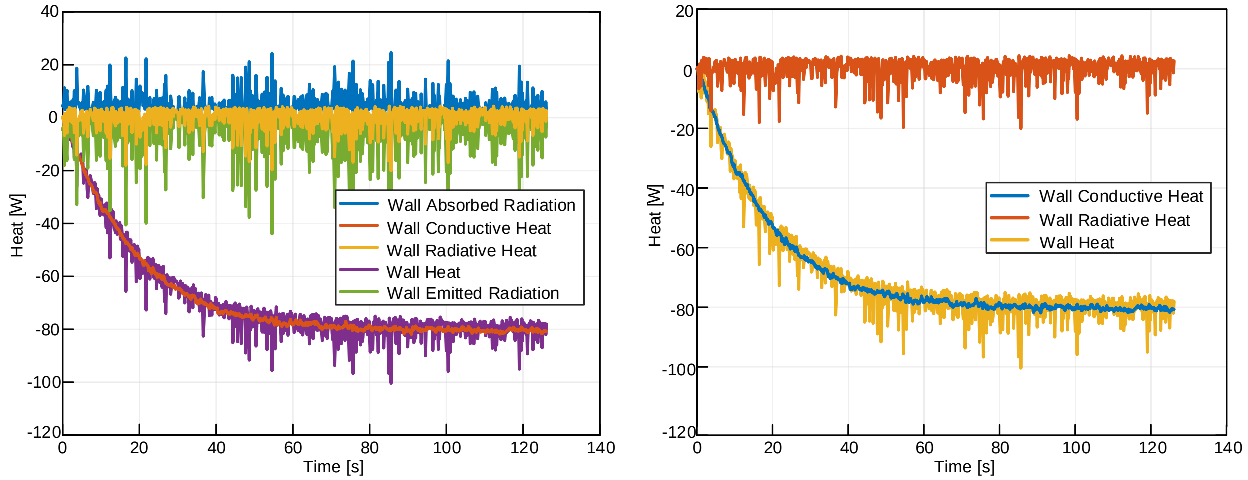

Figure S8. Heat loss investigation results for the outer walls of the IR chamber, showing that the only mode of heat transfer is conduction.

Figure S9 shows the heat loss investigation results for the solid side of the fluid solid interface present on the inner walls of the IR chamber. Here, it can be seen that a steady state is reached very quickly, unlike the outer walls. This is because the assumptions made for the outer walls do not affect the inner walls. There is convection heat transfer present on the inner walls, however convective heat is not shown on the plot since only the solid side of the boundary is being considered. The existence of convection on the inner walls, though it is not shown for the solid side, is likely the reason that the inner walls reach a steady state faster than the outer walls. The inner walls also have the mirror boundary condition, meaning that the radiative heat should be zero. The large spikes and noise that cause the appearance of radiative heat in the plots are the same as those described when discussing the IR chamber outer walls.

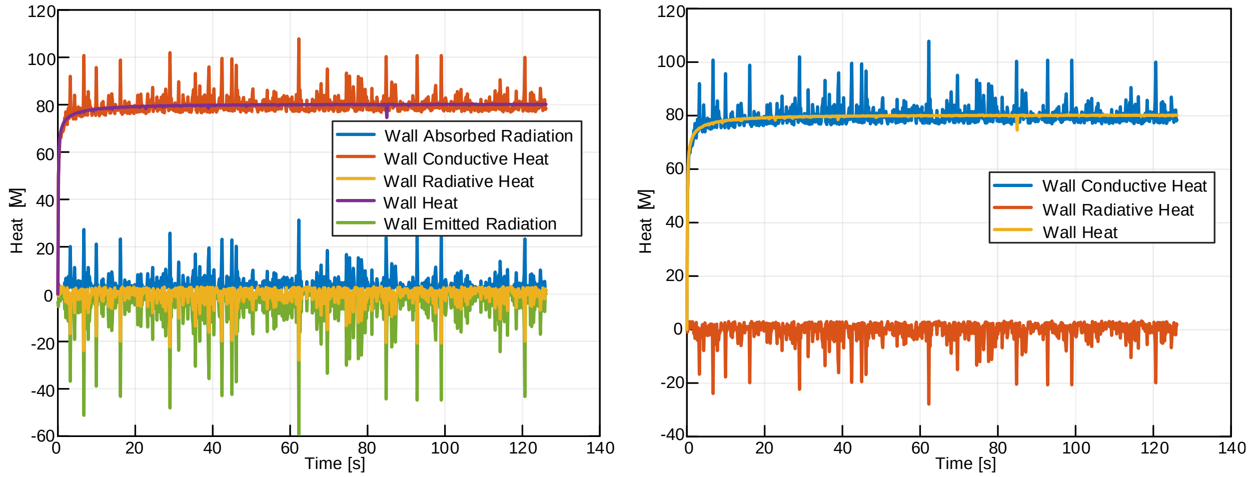

Figure S9. IR chamber inner walls solid side heat loss investigation results.

Figure S10 shows the heat loss investigation results for the fluid side of the IR chamber inner walls. This plot shows that there is convection heat transfer on the inner walls. As described above, the existence of this convective heat is likely the reason for a steady state being reached faster than the outer walls. Here, there is no noise from the radiative heat transfer, and the value is not zero. It can be seen from the plot that the radiative and convective heat added together amounts to the total wall heat.

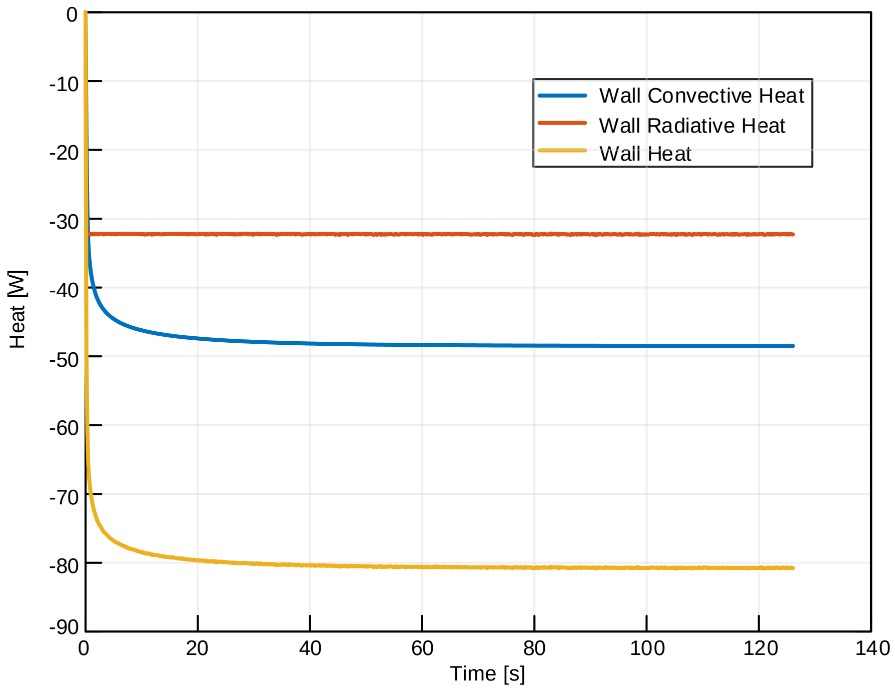

Figure S10. IR chamber inner walls fluid side heat loss investigation results.

Lastly, **Figure S11** shows the heat loss investigation results for the fluid side (left) and solid side (right) of the thermocouple fluid solid interface. On the fluid side, the net heat transfer is negative, due to convection cooling being larger than the radiative heat value. On the solid side, the radiative heat is zero, thus the only mode of heat transfer is conduction. Thus, the solid side dissipates heat through conduction. The line for wall conductive heat matches exactly with the line for wall heat which is the reason it is not seen on the plot.

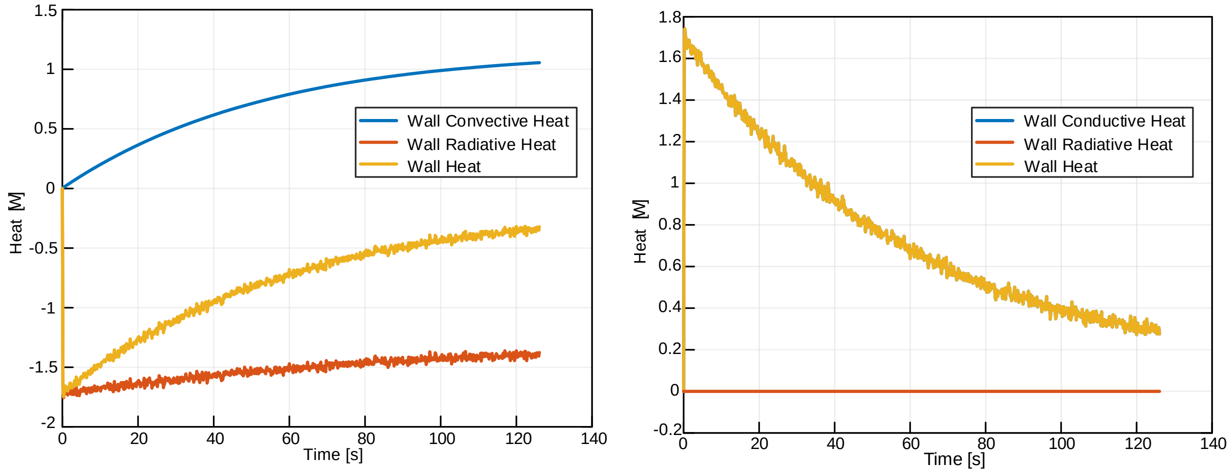

Figure S11. Thermocouple fluid side (left) and solid side (right) heat loss investigation results.

References

(1) Ansys CFX-Solver Theory Guide. **2021**, No. July.

(2) Ansys CFX-Solver Modeling Guide. **2021**.
